# Electric field stimulation boosts neuronal differentiation of neural stem cells for spinal cord injury treatment via PI3K/Akt/GSK-3β/β-catenin activation

**DOI:** 10.1186/s13578-023-00954-3

**Published:** 2023-01-09

**Authors:** Qian Liu, Vsevolod Telezhkin, Wenkai Jiang, Yu Gu, Yan Wang, Wei Hong, Weiming Tian, Polina Yarova, Gaofeng Zhang, Simon Ming-yuen Lee, Peng Zhang, Min Zhao, Nicholas D. Allen, Emilio Hirsch, Josef Penninger, Bing Song

**Affiliations:** 1 Institute of Biomedical and Health Engineering, Shenzhen Institutes of Advanced Technology, Chinese Academy of Sciences, Shenzhen, China; 2 School of Dentistry, College of Biomedical and Life Sciences, Cardiff University, Cardiff, CF14 4XY UK; 3 School of Dental Sciences, Farmington Place, Newcastle University, Newcastle Upon Tyne, NE2 4BW UK; 4 State Key Laboratory of Military Stomatology & National Clinical Research Center for Oral Diseases, School of Stomatology, Fourth Military Medical University, Xi’an, 710032 China; 5 The Brain Cognition and Brain Disease Institute (BCBDI), Shenzhen Institutes of Advanced Technology, Chinese Academy of Sciences, Shenzhen-Hong Kong Institute of Brain Science-Shenzhen Fundamental Research Institution, Shenzhen, China; 6 Bio-X Centre, School of Life Science and Technology, Harbin Institute of Technology, Harbin, 150080 China; 7 State Key Laboratory of Quality Research in Chinese Medicine and, Institute of Chinese Medical Sciences, University of Macau, Macao, China; 8 Department of Ophthalmology and Vision Science, University of California at Davis, Sacramento, CA 95616 USA; 9 School of Biosciences, Cardiff University, Cardiff, CF10 3AX UK; 10 Department of Molecular Biotechnology and Health Sciences, University of Turin, Turin, Italy; 11 Institute of Molecular Biotechnology of the Austrian Academy of Sciences, VBC – Vienna BioCenter, Vienna, Austria; 12 Department of Medical Genetics, Life Sciences Institute, University of British Columbia, Vancouver, Canada

**Keywords:** Neural stem cells, Electric field stimulation, Neuronal differentiation, PI3K/Akt/GSK-3β/β-catenin, Spinal cord injury

## Abstract

**Background:**

Neural stem cells (NSCs) are considered as candidates for cell replacement therapy in many neurological disorders. However, the propensity for their differentiation to proceed more glial rather than neuronal phenotypes in pathological conditions limits positive outcomes of reparative transplantation. Exogenous physical stimulation to favor the neuronal differentiation of NSCs without extra chemical side effect could alleviate the problem, providing a safe and highly efficient cell therapy to accelerate neurological recovery following neuronal injuries.

**Results:**

With 7-day physiological electric field (EF) stimulation at 100 mV/mm, we recorded the boosted neuronal differentiation of NSCs, comparing to the non-EF treated cells with 2.3-fold higher MAP2 positive cell ratio, 1.6-fold longer neuronal process and 2.4-fold higher cells ratio with neuronal spontaneous action potential. While with the classical medium induction, the neuronal spontaneous potential may only achieve after 21-day induction. Deficiency of either PI3Kγ or β-catenin abolished the above improvement, demonstrating the requirement of the PI3K/Akt/GSK-3β/β-catenin cascade activation in the physiological EF stimulation boosted neuronal differentiation of NSCs. When transplanted into the spinal cord injury (SCI) modelled mice, these EF pre-stimulated NSCs were recorded to develop twofold higher proportion of neurons, comparing to the non-EF treated NSCs. Along with the boosted neuronal differentiation following transplantation, we also recorded the improved neurogenesis in the impacted spinal cord and the significantly benefitted hind limp motor function repair of the SCI mice.

**Conclusions:**

In conclusion, we demonstrated physiological EF stimulation as an efficient method to boost the neuronal differentiation of NSCs via the PI3K/Akt/GSK-3β/β-catenin activation. Pre-treatment with the EF stimulation induction before NSCs transplantation would notably improve the therapeutic outcome for neurogenesis and neurofunction recovery of SCI.

**Supplementary Information:**

The online version contains supplementary material available at 10.1186/s13578-023-00954-3.

## Introduction

Neural stem cells (NSCs) are promising candidates for cell replacement therapy in central nervous system (CNS) disorders [1–3] due to their considerable self-renewal and multi-potent differentiation potential into neurons and glia [1, 4, 5]. Many studies, however, have evidenced that the transplanted NSCs exhibit poor survival, neuronal differentiation, and functional maturation due to the pathological niche in spinal cord or brain, regardless of intrinsic genetic modifications or extrinsic growth factors used to promote neuronal differentiation [6–8]. Searching for alternative approaches to enhance survival rate and extent number of mature neurons differentiating from the transplanted NSCs would, therefore, be of great improvement to stem cell transplantation therapies for CNS disorder and injuries.

Along embryonic development, NSCs develop and step to proliferation and differentiation simultaneously with a physiological electric field (EF) at 75–450 mV/mm generated from the trans-epithelial potential and trans-neural tube potential across developing neural tube [9]. Deficiency of the physiological EF artificially would lead to neurodevelopmental disorder and stagnant, indicating a primary role of physiological EF in neural induction, genesis, and development [9, 10]. Our previous studies used the EF stimulation at 300 mV/mm to drive NSCs migration and found the involvement of phosphoinositide 3-kinase (PI3K)/Akt signaling activation [11–13]. However, the questions: how the physiological EF stimulation affect the NSCs proliferation and differentiation? whether the EF stimulated NSCs transplantation would benefit the neural repairment? what is the mechanism of EF stimulation on NSCs fate decision? are still to be explored.

In this study, we applied the physiological EF stimulation at 100 mV/mm to mouse embryonic NSCs to address the effect of EF stimulation on NSCs differentiation. With the NSCs derived from wild type (WT), PI3Kγ^−/−^ (PI3Kγ knock-out) and PI3Kγ^KD/KD^ (PI3Kγ-kinase-dead) mouse embryonic brains, we explored the role of PI3K/Akt/GSK3-β/β-catenin signaling cascade in EF stimulation promoted neuronal differentiation of NSCs. To inspect the translational potent, we then transplanted the physiological EF pre-stimulated NSCs to the spinal cord injury (SCI) mice for impacted neurofunction treatment. The results of this study would lead the way to a better understanding of how stem cell therapy can be optimized by EF stimulation for SCI and other CNS disorders associated with damage or loss of neurons.

## Results

### EF stimulation boosted neuronal differentiation of NSCs

**Fig. 1:**
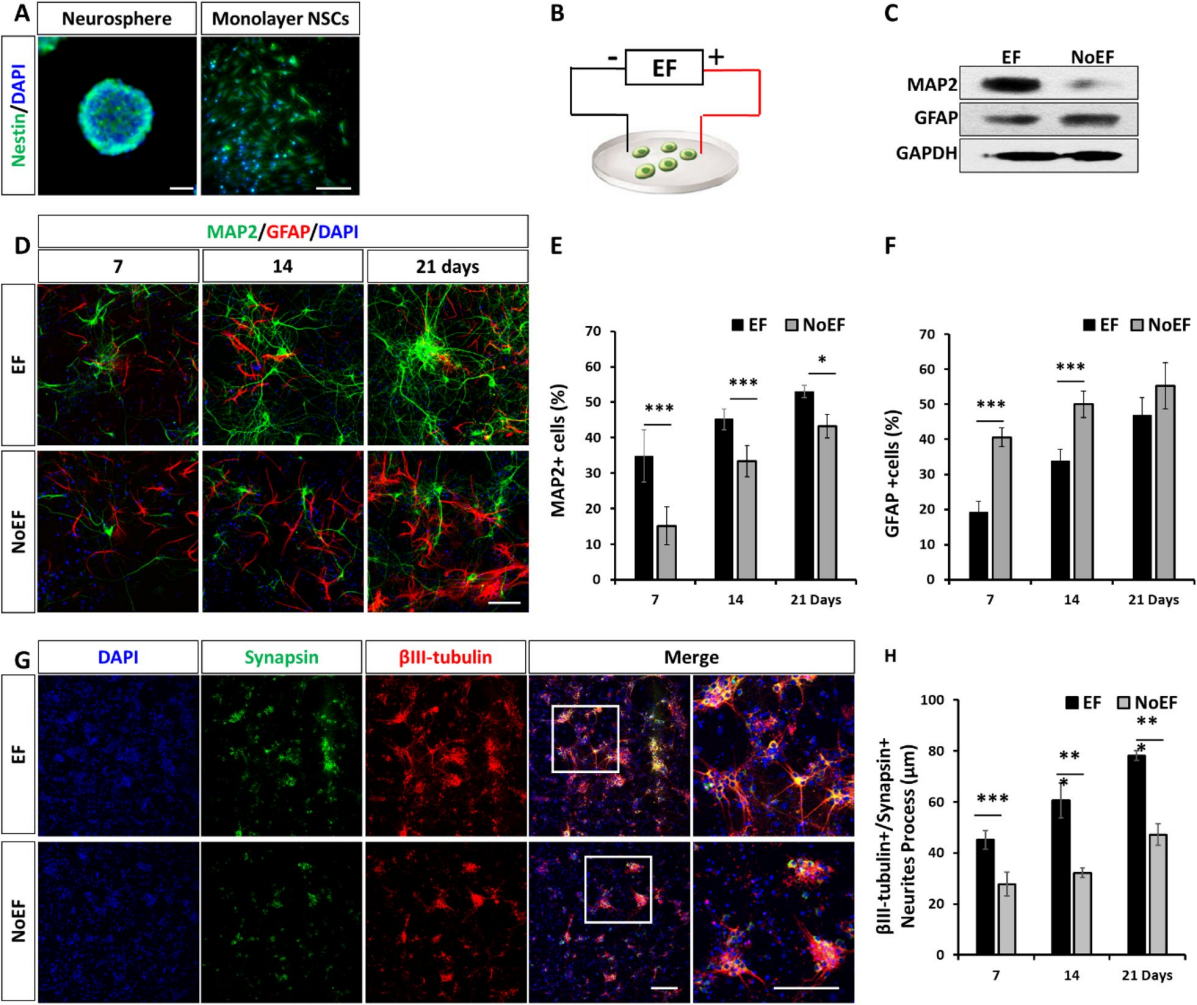
EF stimulation increased neuronal differentiation of NSCs. **A**. Neurospheres and monolayer culture of NSCs for EF stimulation (**B**). **C**. The 7-day EF stimulation increased MAP2 (neuronal differentiation) and decreased GFAP (glial differentiation) protein expression in NSCs. **D**. EF stimulation for 7, 14 and 21 days increased neuronal, and reduced glial differentiation of NSCs. **E**, **F**. Quantification of MAP2 + and GFAP + NSCs counts with/without 7, 14 and 21-day EF stimulation. **G**. EF stimulation improved neurites process (βIII-tubulin + process) and synapse generation (Synapsin +). **H** Quantification of neurites process with/without 7, 14, 21-day EF stimulation. Scale bars: 20 μm. * *P* < 0.05, ** *P* < 0.01 and *** *P* < 0.001 were considered as significantly different between EF and NoEF groups

The NSCs were dissected from the embryonic (E14) C57BL/6 mouse brains and primarily cultured to form neurospheres as published previously ([14, 15], Fig. 1A, left). The primary and sub-cultured NSCs were identified with immunofluorescence, demonstrating a > 95% purity of Nestin-positive NSCs (Fig. 1A). The neurospheres were digested and re-seeded in EF stimulation chamber as described previously [16] to form the monolayer NSCs culture (Fig. 1A, right), for the following EF stimulation procedures (Fig. 1B).

The NSCs were stimulated by a physiological EF at 100 mV/mm, 2 h/day for 7 days in vitro. According to immunoblotting, the neuronal marker of MAP2 was obviously up-regulated, while the glial marker of GFAP was down-regulated, comparing to those cells with non-EF (NoEF) treatment (Fig. 1C). The identical result was also collected with immunofluorescence: the 7-day EF stimulation significantly increased MAP2 in NSCs from 15.2 ± 3.3% to 34.8 ± 1.8% and decreased GFAP from 40.6 ± 2.7% to 19.2 ± 3.0% (Fig. 1D–F). The result indicated the boosted neuronal differentiation with the 7-day EF stimulation induction. Further differentiation through 7 to 14 and 21 days expanded the protein up-regulation of both MAP2 and GFAP, regardless of EF or NoEF treated NSCs. However, on both MAP2 and GFAP, the gaps between EF and NoEF groups gradually shrunk along with the differentiation induction (Fig. 1D–F). Besides, the EF stimulation treatment was also recorded to pro-long the βIII-tubulin + neuronal processes, as well as to increase neuronal synapsin expression (Fig. 1G, H). These results indicated a significantly promoted and accelerated neuronal differentiation of NSCs with the physiological EF stimulation.

### EF stimulation evoked neuronal membrane potential

**Fig. 2:**
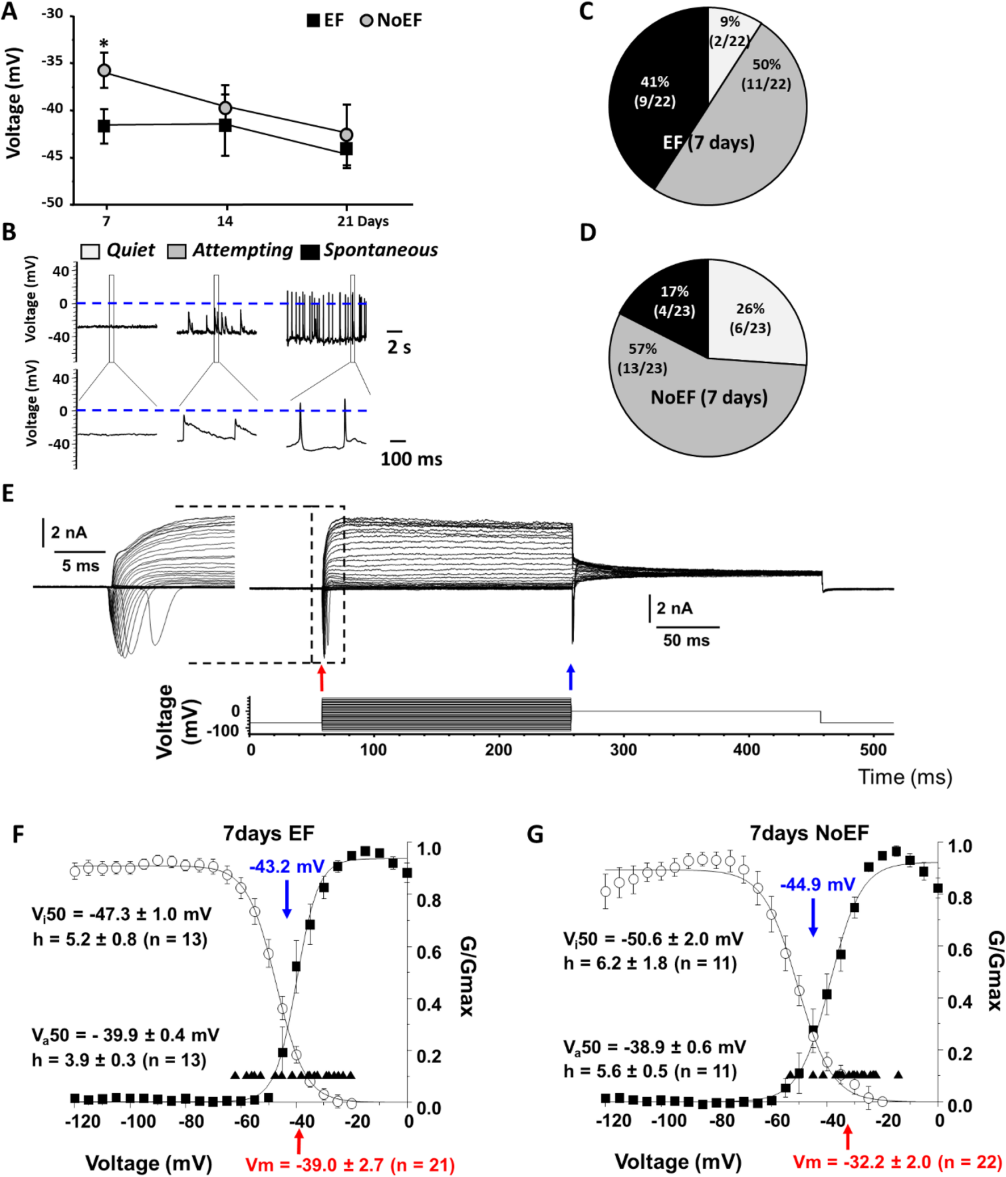
Effect of EF on resting membrane potential (Vm) and voltage-gated Na^+^ current activation and inactivation characteristics of NSCs. **A**. Summary plot of the resting membrane potential (Vm) ontogeny of EF and NoEF treated NSCs at days 7, 14 and 21. Recording were performed in current-clamp. *P < 0.03. **B** Exemplar traces of Vm in NSCs with 7-day EF or NoEF treatment, exhibiting no activity—Quiet (white); Attempting activity with spontaneous action potential-like oscillations below 0 mV (gray); Spontaneous activity with genuine spontaneous action potentials (black). **C**, **D** Pie charts displaying percentage and proportion of various types of neuronal activity (Quiet, Attempting and Spontaneous) in 7-day EF or NoEF, respectively treated NSCs. **E** Exemplar family of whole cell currents (upper lane) during the activation/inactivation voltage protocol (lower lane). Inset (right) illustrate Na^+^ currents. Peak Na^+^ current activation and inactivation levels were shown by the red and blue arrows, respectively. **F**, **G** Mean activation and inactivation characteristics of normalized conductance (G/Gmax) of whole-cell Na^+^ currents recorded in 7-day EF and NoEF treated hiPSC. The activation curves were depicted by the filled squares. The inactivation curves were shown by the empty circles. The individual Vm values were labeled as the filled upward triangles. The mean Vm values were labeled as the red arrow on abscissa in each panel. V_a_50: Voltages of half maximal action. V_i_50: Half maximal inactivation. The h factors: mean crossing points (downward arrows) number of cells recorded for each group (n)

Patch-clamp electrophysiology studies demonstrated that the mean resting membrane potential (Vm) of the 7-day EF stimulation induced NSCs (− 41.7 ± 1.8 mV, n = 22) was significantly more negative compared to the NoEF treated NSCs (− 35.8 ± 1.9 mV, n = 23, *P* < 0.05). At later time-points of the differentiation, 14 and 21 days since the start of EF stimulation, the mean Vm values of EF stimulation and NoEF treated NSCs displayed no statistic difference, suggesting that EF stimulation effect on the functional maturation of NSCs-derived neurons, at an early but not later stage (Fig. 2A). The ability to generate spontaneous action potentials was detected as a functional marker for neuronal differentiation and maturation of NSCs -derived neurons (Fig. 2B). With the 7-day EF stimulation, 41% of the NSCs were boosted to fire action potentials spontaneously (*Spontaneous*; Fig. 2B, C). While as a negative control, 26% of the NoEF treated NSCs exhibited no spontaneous activity (*Quiet*), 57% displayed oscillations of Vm which did not reach 0 mV (*Attempting*), and only 17% exhibited pronounced spontaneous action potentials (*Spontaneous*; Fig. 2B, D).

We also evaluated the EF stimulated neuronal differentiation on human iPSCs. Besides the consistent result on the mean Vm values of EF stimulation and NoEF treated EF 33Qn1 hiPSC-derived NPCs (Additional file 1: Figure S2) as it was shown in the mouse NSCs, the activation/inactivation characteristics of the voltage-activated Na^+^ currents in neurons displayed progressive increase of the normalized conductance (G/Gmax) maxima during neuronal maturation (weeks 1–3 of in vitro culturing) in both EF and NoEF groups. The mean resting Vm of EF neurons consistently had more negative values compared to noEF neurons in the timing pairs, which suggests of faster maturation rate of EF 33Qn1 hiPSC-derived NPCs (Fig. 2E, G). As we have shown earlier the effect of significant increase of Vm in the human iPSC-derived NSC undergoing EF stimulation is determined by augmented expression of Kv7 channels [17].

To summarize, the electrophysiology results suggested that EF stimulation enhanced functional maturation of NSCs by two major biophysical enhancements: by hyperpolarizing the cells to withdraw inactivation of voltage-gated Na^+^ channels which enables their higher spontaneous activity, and by increasing the Na^+^ current availability, to facilitate regenerative action potential Train activity [18].

### EF stimulation induced PI3K/Akt/GSK-3β/β-catenin activation

**Fig. 3:**
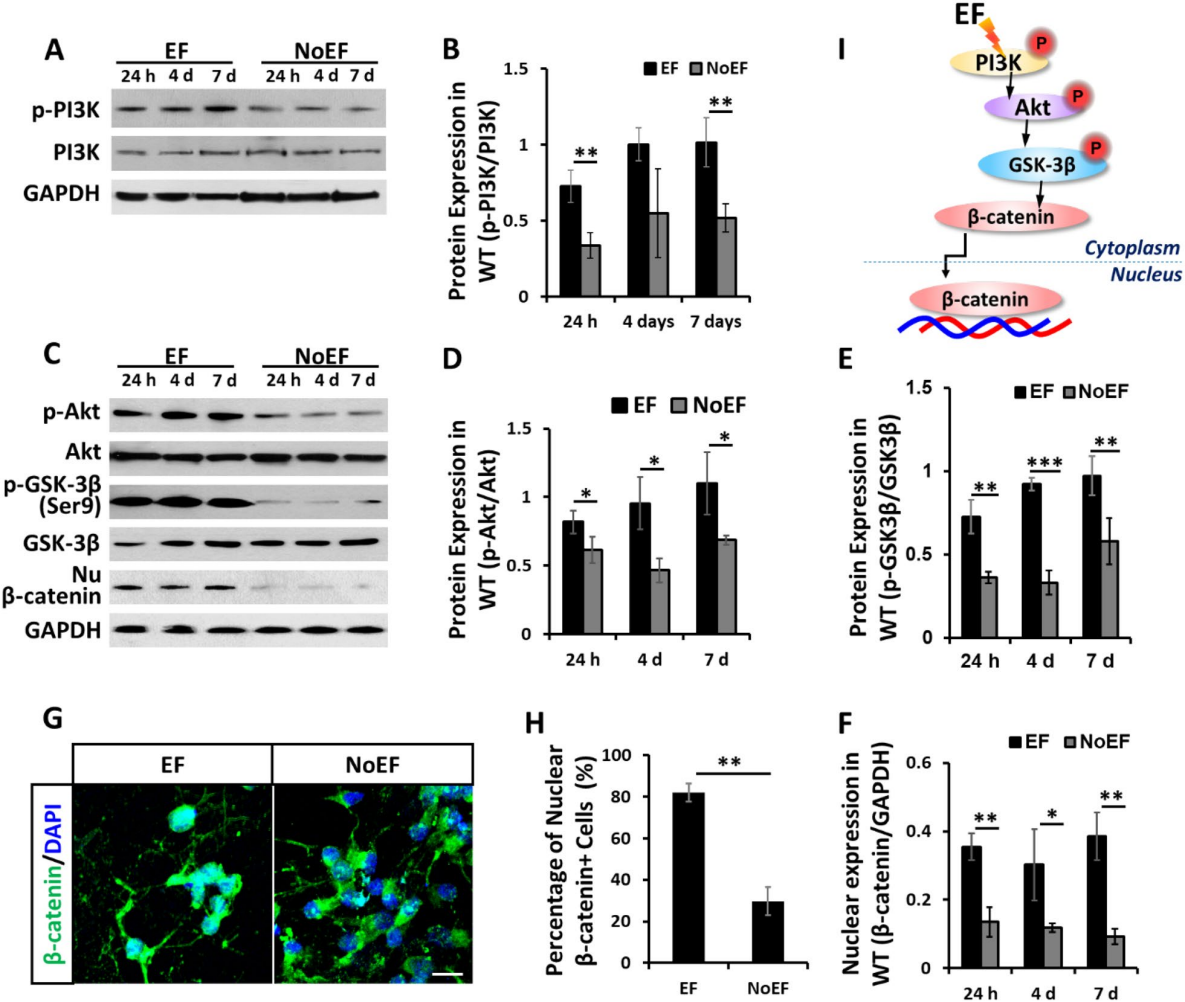
EF stimulation activated PI3K/Akt/GSK-3β/β-catenin pathway in NSCs. **A**, **B** EF stimulation increased phosphorylation of PI3K. **C**–**F**. EF stimulation increased phosphorylation of Akt and GSK-3β at Ser9, and expression of β-catenin in NSC nuclei. **G**, **H**. EF stimulation increased nuclear translocation of β-catenin. **I**. Schematic diagram of EF stimulation induced PI3K/Akt/GSK-3β/β-catenin pathway activation in NSCs. Scale bar: 10 μm. * *P* < 0.05 and ** *P* < 0.01 were considered as significantly different between EF and NoEF groups

We then explored the involved intracellular signaling mechanism of the EF boosted neuronal differentiation of NSCs. According to protein expression and phosphorylation detection by immunoblotting, the p-PI3K/PI3K ratio was significantly increased by EF stimulation, demonstrating an up-regulated PI3K activation (Fig. 3A, B). The activation by EF stimulation was then transduced to the down-streaming Akt and GSK-3β at Ser9, manifesting as the increased ratios of p-Akt/Akt and p-GSK-3β (Ser9)/ GSK-3β (Fig. 3C–E). As a down-streaming signal of GSK-3β (Ser9) activation, according to both immunoblotting (Fig. 3C, F) and immunofluorescence (Fig. 3G, H), we detected the significantly increased expression of β-catenin in nucleus with the 24 h, 4d and 7d EF stimulation. The result indicated an enhanced nuclear translocation of β-catenin by EF stimulation. In summary, the EF stimulation triggered activation of PI3K/Akt/GSK-3β/β-catenin cascade in NSCs (Fig. 3I).

### Deficiency of PI3Kγ or β-catenin abolished the boosted neuronal differentiation of NSCs by EF stimulation

**Fig. 4:**
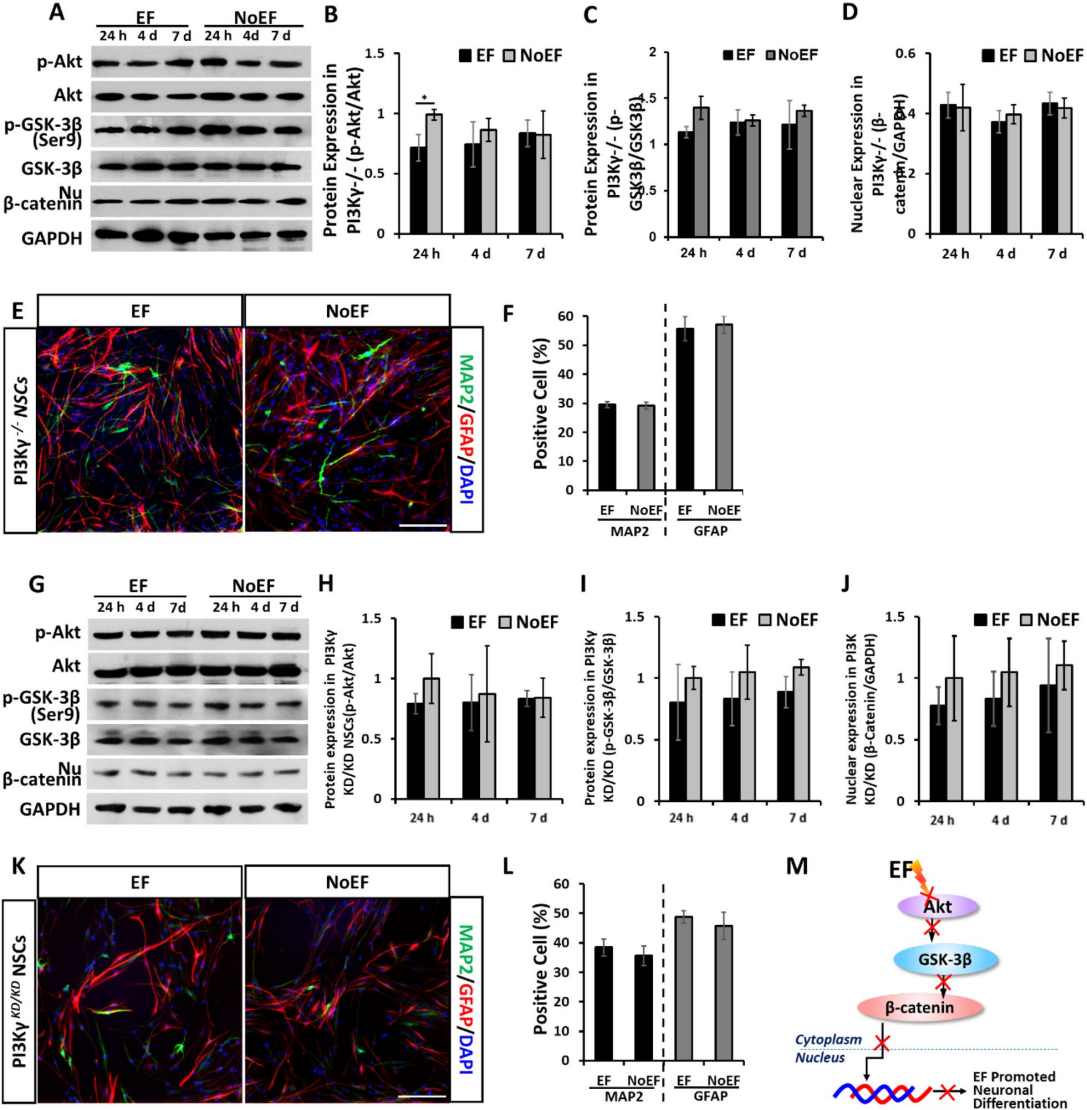
PI3Kγ deficiency abolished the PI3K/Akt/GSK-3β/β-catenin activation and neuronal differentiation of NSCs induced by EF stimulation. **A**–**D**. EF stimulation failed to increase the Akt and GSK-3β (Ser9) phosphorylation and β-catenin nuclear expression in PI3Kγ^−/−^ NSCs. **E**–**F**. EF stimulation showed no effect on expression of MAP2 or GFAP in PI3Kγ^−/−^ NSCs. **G**-**J**. EF stimulation failed to increase the Akt and GSK-3β (Ser9) phosphorylation and β-catenin nuclear expression in PI3Kγ^KD/KD^ NSCs. **K**, **L**. EF stimulation showed no effect on expression of MAP2 or GFAP in PI3Kγ^KD/KD^ NSCs. **M**. Schematic diagram: PI3Kγ was required in EF stimulation induced PI3K/Akt/GSK-3β/β-catenin pathway activation and neuronal differentiation in NSCs. Scale bars: 20 μm. * *P* < 0.05 was considered as significantly different between EF and NoEF groups

To further address the role of PI3K/Akt/GSK-3β/β-catenin cascade in EF stimulation promoted neuronal differentiation of NSCs, we then apply EF stimulation on the PI3Kγ^−/−^ NSCs derived from embryonic PI3Kγ^−/−^ mouse brains [19] and the PI3Kγ^KD/KD^ NSCs derived from embryonic PI3Kγ-kinase-dead mouse brains [20]. When either PI3Kγ expression (Fig. 4A–F) or its kinase activity (Fig. 4G–L) was deficient, the down-streaming Akt and GSK-3β were silenced to the 24 h, 4d and 7d EF stimulation. In PI3Kγ^−/−^ NSCs, EF stimulated p-Akt/Akt at 24 h (Fig. 4B) and p-GSK-3β (Ser9)/GSK-3β at 7d (Fig. 4C) were even detected decreased than that in the NoEF group. The effect of EF stimulation to increase the nuclear expression of β-catenin was also abolished in either PI3Kγ^−/−^ or PI3Kγ^KD/KD^ NSCs, from 24 h, 4d through 7d (Fig. 4A, D, G, J) . Along with the silence of PI3K/Akt/GSK-3β/β-catenin signaling to EF stimulation in both PI3Kγ^−/−^ and PI3Kγ^KD/KD^ NSCs, no statistical difference was detected on either MAP2 or GFAP expression between the 7-day EF and NoEF treated groups (Fig. 4E, F, K, L). In summary, the result indicated the requirement of PI3Kγ and its kinase activity, in EF stimulation activated PI3K/Akt/GSK-3β/β-catenin and boosted neuronal differentiation of NSCs (Fig. 4M).

**Fig. 5:**
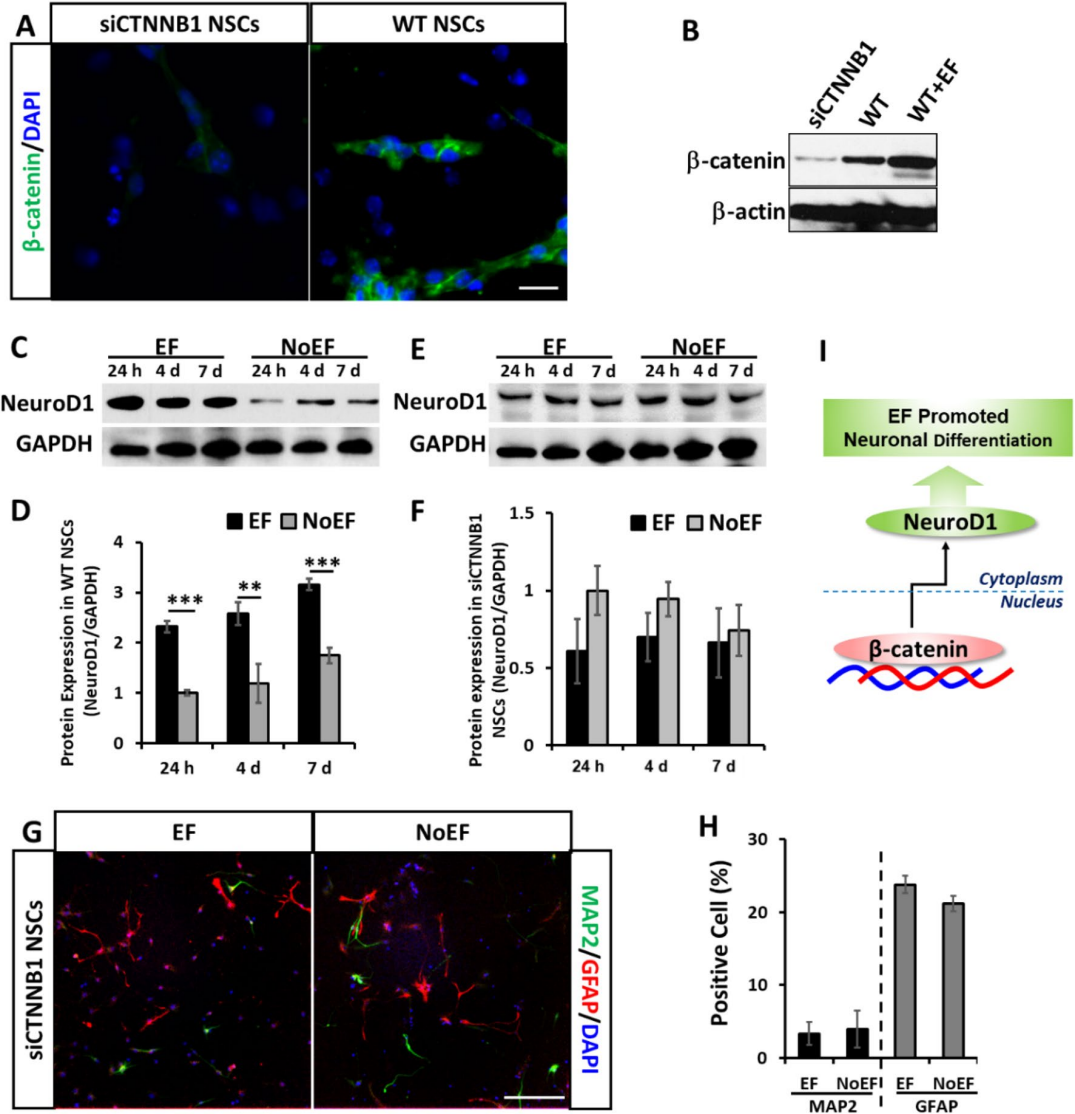
Knock down of β-catenin abolished the neuronal differentiation of NSCs induced by EF stimulation. **A**, **B** The β-catenin expression in NSCs was knocked down with siCTNNB1 transfection. Scale bar: 10 μm. **C**, **D** EF stimulation increased NeuroD1 in WT NSCs. **E**, **F** EF stimulation failed to increase (or even in reversing trend) NeuroD1 expression in siCTNNB1 transfected NSCs. **G**, **H**. EF stimulation showed no effect on expression of MAP2 or GFAP in siCTNNB1 transfected NSCs. Scale bar: 20 μm. **I**. Schematic diagram: β-catenin is required in EF stimulation induced the NeuroD1 and neuronal differentiation of NSCs. * *P* < 0.05, ** *P* < 0.01 and *** *P* < 0.001 were considered as significantly different between EF and NoEF groups

As one of down-streaming effectors of β-catenin nuclear translocation, also a neuronal differentiation marker of NSCs, NeuroD1 was recorded significantly up-regulated by the 24 h, 4d and 7d EF stimulation (Fig. 5C). Whilst, in siCTNNB1 siRNA transfected NSCs (β-catenin knock-down) (Fig. 5A, B), the up-regulation of NeuroD1 by EF stimulation was abolished, and even reversed to express less than that in NoEF treated group (Fig. 5E, F). Consistent with the results from PI3Kγ^−/−^ and PI3Kγ^KD/KD^ NSCs (Fig. 4), no statistical difference was detected on MAP2 or GFAP expression between 7-day EF and NoEF treated groups when β-catenin was knock-down by siCTNNB1 transfection (Fig. 4G, H). The results recommended the essential decision role of β-catenin nuclear translocation in EF stimulated differentiation of NSCs (Fig. 5I), while the PI3K/Akt/GSK-3β activation was its up-streaming inducting signal.

### EF pre-stimulation improved neurogenesis of transplanted NSCs in impacted spinal cord

**Fig. 6:**
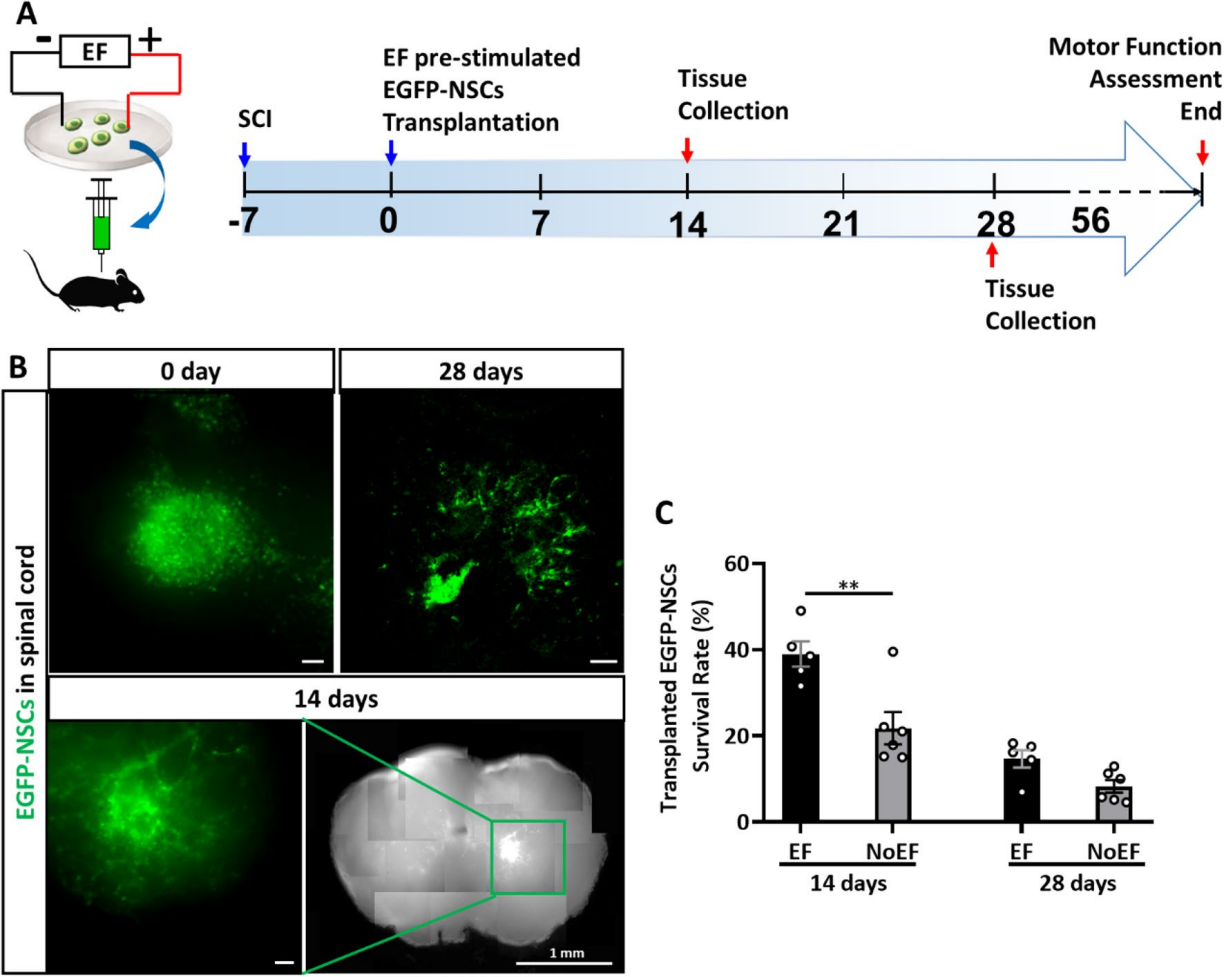
EF stimulated NSCs transplantation for spinal cord injury. **A**. SCI surgery and NSCs transplantation design. **B**. EF pre-stimulated EGFP-NSCs expanded from the injection site after transplanted into the impacted spinal cord for 0, 14 and 28 days. Scale bars for the EGFP images: 20 μm; Scale bar for the spinal cord map: 1 mm. **C**. EF pre-stimulation promoted higher NSCs survival percentage (EGFP-NSCs count/injected 10^5^ cells X 100%) in the injured spinal cord, comparing to the NoEF NSCs transplantation group. * *P* < 0.05 and ** *P* < 0.01 were considered as significantly different between EF and NoEF groups. n = 5 mice in EF and n = 6 mice in NoEF group

To further confirm the effect of EF stimulation on boosting neuronal differentiation of NSCs, as well as to improve the stem cell transplantation efficiency for CNS disorders, we transplanted the EF pre-stimulated NSCs for the SCI modeling mice treatment (Fig. 6A).

To track the transplanted cells in spinal cord, we cultured in vitro, EF pre-stimulated and transplanted the EGFP-NSCs derived from embryonic C57BL/6-Tg(CAG-EGFP)10sb/J mouse brains. According to the EGFP fluorescence in the transverse spinal cord slices, the injected EGFP-NSCs in spinal cord was detected radially diffused since 0 through 28 DAT (days after transplantation) (Fig. 6B). Immunofluorescence of EGFP recorded 40 ± 6.6% (n = 5 mice in EF group) survival rate of the EF pre-stimulated EGFP-NSCs in the impacted spinal cord 14 DAT, significantly higher than that of the NoEF treated EGFP-NSCs transplantation group with the survival rate at 21.9 ± 9.1% (*P* = 0.0017, n = 6 in NoEF group). The survival rate difference between the two groups reduced at 28 DAT (Fig. 6C). The results recommended that the EF pre-stimulation benefited the transplanted stem cell survival in hosting spinal cord tissue, especially at early stage (0–14 DAT) of the transplantation for spinal cord injury treatment.

**Fig. 7:**
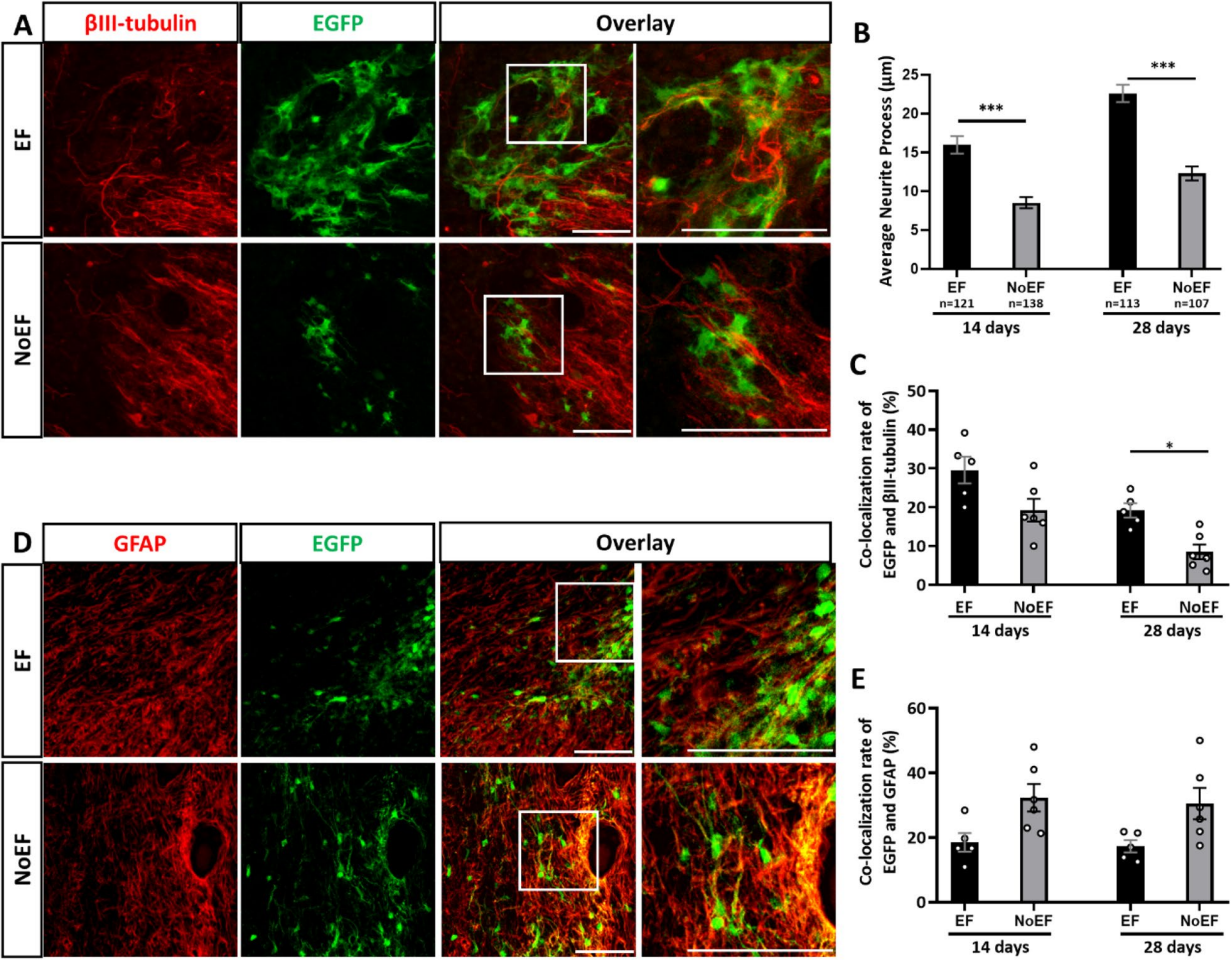
EF pre-stimulation increased neuronal differentiation of transplanted NSCs in impacted spinal cord. **A**–**C**. The transplanted EGFP-NSCs with EF pre-stimulation showed increased neurite process (**A**, **B**) and βIII-tubulin/EGFP co-localization (**A**, **C**) than those NSCs with NoEF treatment **D**, **E**. The transplanted EGFP-NSCs with EF pre-stimulation showed less GFAP/EGFP co-localization than those NSCs with NoEF treatment. Scale bars: 20 μm. * *P* < 0.05, ** *P* < 0.01 and *** *P* < 0.001 were considered as significantly different between EF and NoEF groups. n = 5 mice in EF and n = 6 mice in NoEF group

The following question was the effect of EF pre-stimulation on long-term differentiation of the transplanted NSCs in the impacted spinal cord. To address the question, we dissected and sliced the SCI mouse spinal cord with EF pre-stimulated or NoEF treated NSCs transplantation, for immunofluorescence at 28 DAT (Fig. 7A, D). According to the EGFP + neurite process measurement, the EF pre-stimulation pro-longed the neurite growth at both 14 (*P* < 0.001) and 28 (*P* < 0.001) DAT (n = 5 mice in EF and n = 6 mice in NoEF group, Fig. 7B). Although a higher ratio of EGFP/βIII-tubulin (Fig. 7C) and lower of EGFP/GFAP (Fig. 7E) co-localization with EF pre-stimulation at 14 DAT were detected, there was no statistic differentiation between the EF and NoEF groups. Later stage at 28 DAT, as the proportion of EGFP/βIII-tubulin co-localization from both groups all dropped, the EF pre-stimulated NSCs turned out with significantly higher count than that of the NoEF treated NSCs (*P* = 0.0452, n = 5 mice in EF and n = 6 mice in NoEF group, Fig. 7C). Whist, for the EGFP/GFAP co-localization, no difference was detected between 14 and 28 DAT, neither between EF nor NoEF groups (Fig. 7E).

### EF pre-stimulated NSCs transplantation benefited neurogenesis and hind limb motor function recovery of spinal cord injured mice

Besides the boosted neuronal differentiation of the transplanted NSCs, we further explored the neurogenesis and neurofunctional recovery of the SCI mice, receiving either EF pre-stimulated or NoEF treated NSCs transplantation.

**Fig. 8:**
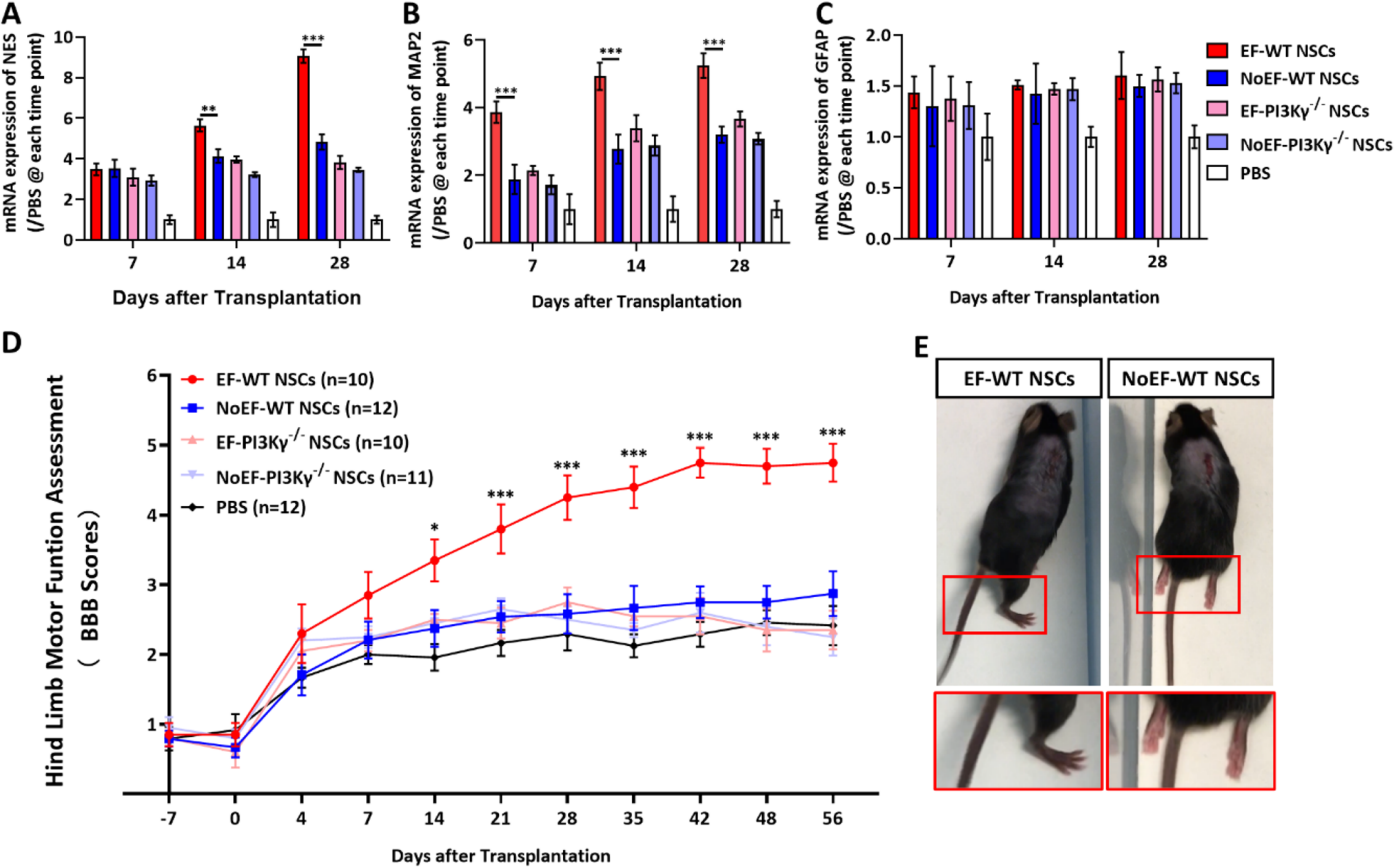
EF pre-stimulated NSCs transplantation improved neurogenesis and motor function recovery of spinal cord injury. **A**–**C**. The mRNA expressions of NES (nestin, NSCs marker), MAP2 (neuronal differentiation) and GFAP (glial differentiation) from the spinal cord of the SCI mice with NSCs transplantation. **D** BBB assessment (average score of right and left hind limbs) for hind limb motor function recovery of the SCI mice with NSCs transplantation. The mice with EF pre-stimulated WT NSCs transplantation demonstrated better motor function repairmen than NoEF WT NSCs. Whilst, with the PI3Kγ^−/−^ NSCs, the effect of EF pre-stimulation was abolished. A group with PBS injection was used as a negative control. **E** Exemplar photos of SCI mice with EF and NoEF pre-stimulated NSCs transplantation, 28 DAT. The SCI mouse receiving EF pre-stimulated NSCs transplantation showed occasional weight-supported dorsal stepping on right hind limb (left). While the SCI mouse receiving NoEF treated NSCs transplantation showed slight movement of both hind limbs, without any weight-support. * *P* < 0.05, ** *P* < 0.01 and *** *P* < 0.001 were considered as significantly different between EF and NoEF groups

For neurogenesis, the spinal cord tissues receiving SCI impact and NSCs transplantation from each experimental group were collected for mRNA expression analysis. As a marker of neurogenesis, the mRNA expression of NES (Nestin) of the spinal cord tissue was detected significantly up-regulated with the EF pre-stimulated NSCs transplantation (EF-WT NSCs vs. NoEF-WT NSCs), from 14 (*P* = 0.0068, n = 3 mice for each group) through 28 (P < 0.001, n = 3 mice for each group) DAT (Fig. 8A). For the neuronal marker expression of MAP2, the mRNA expression was also detected significantly up-regulated with the EF pre-stimulated NSCs transplantation even earlier, from 7 through 28 DAT (*P* < 0.001 for the three time points, n = 3 mice for each group Fig. 8B). While, for the glial marker of GFAP, no mRNA expression difference was detected between the EF pre-stimulated and NoEF treated WT NSCs transplantation groups (Fig. 8C). When the PI3Kγ^−/−^ NSCs was used for EF pre-stimulation and transplantation for SCI treatment, neither difference of NES, MAP2 or GFAP was detected from the group of mouse spinal cord slices, receiving EF pre-stimulated or NoEF treated PI3Kγ^−/−^ NSCs transplantation (n = 3 mice for each group, Fig. 8A–C). The results indicated the improving effect of the EF pre-stimulated NSCs transplantation on the neurogenesis post SCI, especially for the neuronal genesis, as well as to confirm the essential role of PI3K in EF stimulation induced NSCs fate decision for neurogenesis in SCI.

The neurofunctional recovery of the SCI mice receiving NSCs transplantation was assessed with the Basso, Beattie, and Bresnahan (BBB) scores of bilateral hind limbs [21]. Comparing with the group receiving PBS injection as a negative control (n = 12 mice), the SCI mice with EF pre-stimulated WT NSCs transplantation (n = 10 mice) performed significantly higher BBB scores, from 14 through 56 DAT. While the NoEF treated WT NSCs transplantation (n = 12 mice) group showed no sign of better recovery to the PBS group (Fig. 8D, E). When the PI3Kγ^−/−^ NSCs was used for EF pre-stimulation and transplantation for SCI treatment, no matter with or without the EF pre-stimulation, the PI3Kγ^−/−^ NSCs transplantation was recorded no effect on BBB score increase than PBS group (Fig. 8D). The results indicated a benefited neurofunctional recovery of SCI with the EF pre-stimulated NSCs transplantation cue. Moreover, the beneficial effect from the transplanted NSCs responding to EF pre-stimulation required PI3K as a potential mediating signal.

## Discussion

When stem cell transplantation is used for CNS disease treatment, four major factors should be addressed: survival of the transplanted cells; proliferation and self-renew; migration to the target site; and differentiation into functional mature cells. In our previous studies, we have revealed the controlling effect of EF stimulation on directing the NSCs migration [11], which recommended a potential approach to recruit the transplanted NSCs at the lesion site for neurological recovery. Although assembled at the lesion site, most of these transplanted NSCs might be induced to glial rather than neuronal differentiation, by the pathological micro-environment. Therefore, the neuronal cell replacement efficiency is always reported lower than theoretically expected. Finding approaches to improve neuronal differentiation would help in the development of more effective stem cell therapies for CNS diseases. In this study, we boosted the neuronal differentiation of NSCs with physiological EF stimulation, explored the underlying signaling mechanism and transplanted the EF pre-stimulated NSCs for SCI treatment.

One of three major findings of our study was the boosted neuronal differentiation of NSCs by physiological EF stimulation, demonstrated with the increased MAP2 + cells, decreased GFAP + cells and the elongated neurite process (Fig. 1). We also recorded the accelerated and enhanced neuronal maturation, demonstrated by a significant augmentation of neuronal resting membrane potential (Vm) and increased percentage of neurons exhibiting spontaneous activity (Fig. 2 and Additional file 1: Fig. S2). EF-dependent increases in Vm and therefore the withdrawal of Na^+^ channel inactivation enables the neurons to fire, which suggested the up-regulation of Kv7 (M)-channels responsible for the maintenance of Vm and the regulation of neuronal excitability [22]. Besides augmented expression of Kv7 channel subunits [17] EF-induced physiological up-regulation of Kv7-channels might employ the PI3K signaling with subsequent increase of endogenous PIP3 concentration, which has been shown to be more effective than PIP2 for Kv7-channel stimulation [23]. These data demonstrate that EF stimulation boosts up-regulation of factors responsible for the rate and extent of neuronal maturation.

To explore the mechanism of EF boosted neuronal differentiation of NSCs, we studied the candidate signaling networks. Our previous work have demonstrated that PI3K is required in EF-directed cell migration and wound healing [24], specifically the EF-directed migration and proliferation of NSCs [11]. The PI3K/Akt pathway has also been reported as a primary regulator of stem cell fate decision [15, 25, 26]. The role of PI3K/Akt in EF-enhanced neuronal differentiation of NSCs, however, has yet been clearly explored. This study provided the evidence that EF promoted neuronal differentiation of NSCs, requiring PI3K activation via the functional catalytic subunit, PI3Kγ. Either genetically knockout of PI3Kγ [19], or transgenically block the kinase activity of PI3Kγ [27] was recorded significantly suppressed, or even reversed the EF boosted neuronal differentiation of NSCs (Fig. 4). The transplantation with PI3Kγ^*−/−*^ NSCs in SCI mice also confirmed the requirement of PI3Kγ in EF promoted neuronal differentiation of the transplanted NSCs (Fig. 7), which was confirmed to benefit the neurogenesis and neurological function recovery post SCI (Fig. 8).

GSK-3β is one of the major down-streaming signals of PI3K/Akt activation, which would be phosphorylated at Ser9 when the activation is transduced from PI3K through Akt [28–30]. It is also well documented that GSK-3β is involved in Wnt/β-catenin controlled NSCs differentiation, playing a role to regulate β-catenin either to be degraded or to be released for nuclear translocation and trigger the down-streaming gene transcription [31–33]. In this study, we found that EF stimulation triggered GSK-3β phosphorylation at Ser9 following PI3K/Akt activation and increased the nuclear translocation of β-catenin. When the activation signal by EF was blocked in PI3Kγ^−/−^ or PI3Kγ^KD/KD^ NSCs, both GSK-3β (Ser9) phosphorylation and β-catenin nuclear translocation were abolished, therefore leading to less neuronal differentiation of PI3Kγ^−/−^ or PI3Kγ^KD/KD^ NSCs, even by EF stimulation (Fig. 4). The results suggested a switching role of GSK-3β (Ser9) in mediating the signals from PI3K/Akt to Wnt/β-catenin, which control the neuronal differentiation of NSCs by EF.

We then further studied β-catenin as a transcription factor and its following effector, NeuroD1 in EF stimulated neuronal differentiation of NSCs. As discussed above, β-catenin nuclear translocation was triggered by EF stimulation through PI3K/Akt/GSK-3β (Ser9) activation, leading to the up-regulated transcription of NeuroD1. When β-catenin was knockdown with CTNNB1-specific siRNA (siCTNNB1) transfection, the down-stream NeuroD1 as a neuronal differentiation marker gene, was detected silent to the EF stimulation. The siCTNNB1 NSCs were also detected failed in EF stimulated neuronal differentiation (Fig. 5). Taken together the results from PI3Kγ and β-catenin deficient NSCs responding to the EF stimulation, we explain the mechanism of EF stimulation promoted neuronal differentiation with the signal transduction through PI3K/Akt/GSK-3β/β-catenin cascade activation.

Improved neurofunction recovery have been previously reported when stem cells were transplanted for SCI treatment. For the host neuron in spinal cord tissue, serials of reported studies have evidence the neuronal damage, apoptosis and necrosis by SCI pathologies and glial scar formation in the acute phage of SCI; while the neurogenesis, regeneration and neuroplasticity happen in the chronic phage [34–36]. However, the SCI pathological cytokines, immune and inflammatory factors produce a very terrible niche for local neuron survival and regeneration, therefore an endogenous neural stem/progenitor cell transplantation would contribute as a regeneration pool, neural growth/trophic factor and anti-inflammatory support [4]. However, induced by the pathological factors, such as inflammatory and immune factors at the lesion site, the transplanted stem cells were proved more likely to be driven to glial differentiation [37]. While in our study, the physiological EF stimulation was confirmed to promote neuronal, rather than glial differentiation of NSCs, through PI3K/Akt/GSK-3β cascade activation. We then transplanted these EF pre-stimulated NSCs to SCI mice to study the cell fate of transplanted NSCs in spinal cord, as well as the neurofunction recovery of SCI mice. Within the first 14 DAT, the transplanted NSCs were detected more mobilized by EF pre-stimulation, evidenced with expanded diffusion, higher remain and survival rate (EGFP cell remain 14 DAT) and longer neurite process (Fig. 6B, C, Fig. 7B). However, no significant neuronal or glial differentiation was detected in this stage, either with or without EF pre-stimulation (Fig. 7C–E). As for the receiving host SCI mice in the stage, although the induced neurogenesis with higher NES (Nestin) and MAP2 mRNA expressions in the injured spinal cords were detected with EF pre-stimulated WT NSCs transplantation (Fig.7A, B), the BBB scores representing neurofunction recovery did not show statistic difference at 7 and 14 DAT (Fig.7C). While for the longer-term investigation at 28 DAT, less remaining NSCs (EGFP + cell count) were detected from the injured spinal cord, no matter with or without EF pre-stimulation (Fig. 6C). While significantly longer neurite process (Fig. 7B) and neuronal differentiation (Fig. 7C) by EF pre-stimulation were the cell fate character for the transplanted NSCs at this stage. At the same time, the receiving host SCI mice demonstrated the significantly improved neurogenesis (Fig. 8A, B) and neurofunction recovery (Fig. 8D) from 14 through 28 DAT with EF pre-stimulated WT NSCs transplantation. When thePI3Kγ^−/−^ NSCs were used for transplantation, either with or without EF pre-stimulation, the improving effects on neurogenesis in the injured spinal cord and neurofunction recovery of the SCI mice were both abolished (Fig. 8). In summary of the in vivo results, the EF pre-stimulation promoted the mobilization, remain and survival of the transplanted NSCs at early stage of 14 DAT and the neuronal differentiation at later stage of 28 DAT; the EF pre-stimulated NSCs transplantation improved the neurogenesis of the injured spinal cord and neurofunction recovery of the SCI mice, especially for the later stage when the promoted neuronal differentiation were detected starting to amplify; PI3Kγ activity was required in EF pre-stimulated NSCs transplantation for SCI treatment. We assume that the significant increased the short-term (14 DAT) survival of transplanted NSCs by EF pre-stimulation than NoEF transplantation (Fig. 6C) would produce more cell-to-cell support and anti-inflammatory effect, therefore produce more support to neuronal rather than glial differentiation, despite of the SCI pathological niche. When lasting as long-term as 28 DAT or even further, although the EF pre-stimulated NSCs also dropped, it produced significant higher neuronal (MAP2 + , Fig. 7C, 28 DAT) and less glial (GFAP + , Fig. 7E, 28 DAT) differentiation. In summary, the survival rate of transplanted NSCs contribute to the following neurogenesis and differentiation; while the induced upper ratio of neuron:glia by EF pre-stimulation contributed mainly to the long-term neurofunction recovery assessed by BBB scores.

In conclusion, our study confirmed physiological EF stimulation at 100 mV/mm as a promising method to boost neuronal differentiation of NSCs, through the activation of PI3K/Akt/GSK-3β/β-catenin cascade. The EF pre-stimulation improved the survival and differentiation of the transplanted NSCs in impacted spinal cord, as well as benefited the neurogenesis and neurofunction recovery post SCI. In different stage post transplantation, stem cell survival, neuronal differentiation, neuron:glial ratio contribute to the tissue regeneration and neurofunction recovery. Our EF pre-stimulation method could promote the NSCs survival and neuronal differentiation, therefore promote the stem cell treatment for SCI in both short- and long-term therapeutic outcome. The findings of this study would lead the way to a better understanding of how stem cell therapy can be optimized by EF stimulation for SCI and other CNS disorders associated with damage or loss of neurons.

## Materials and methods

### Animals

All experiments were carried out in accordance with the Animals (Scientific Procedures) Act 1986 under project licenses 30/2816 issued by UK Home Office. This study was approved by the Cardiff University and Shenzhen Institutes of Advanced Technology (SIAT), Chinese Academy of Sciences Research Ethics Committees. The mice were housed in a temperature-controlled environment (22 ± 0.5 ℃) with a 12-h-light–dark cycle and allowed free access to food and water. All efforts were made to minimize animal suffering and reduce the number of animals used. Spinal cord injury (SCI) surgery was administrated on adult female mice (20–22 g) in this study. The EF/NoEF treated NSCs were injected into the spinal cord on both rostral and caudal sides to the lesion point 7 days after the SCI surgery. The neurological function of mice post SCI surgery was assessed according to the BBB score system as described previously [21]. The mice were sacrificed at day 14 and 28 post NSC transplantation for spinal cord tissue collection. The in vivo samples were subjected to further real-time PCR and immunofluorescence analysis**.**

### Neural stem cells (NSCs) and human pluripotent stem cells (hiPSCs)

The Wild type, EGFP labelled, PI3Kγ^−/−^ and PI3Kγ^KD/KD^ NSCs were dissected from embryonic brain tissue of E14 day WT C57BL/6 mice, EGFP-actin (G57BL/6-Tg(CAG-EGFP)10sb/J mice shared by Dr Ketan Patel from University of Reading, UK. Jackson laboratory, Stock No.: 003291), Pik3cg^–/–^ (Pik3cg^tm1Pen^ mice, provided by Dr Josef Penninger [19]) and PI3Kγ-kinase-dead (KD) (Pik3cg^tm1Ehi^ mice, provided by Dr Emillio Hirsch [20]) mice, using the neurosphere method as described previously [11, 38]. For monolayer culture of the NSCs, the neurospheres were digested into single cell suspension by Accutase (Invitrogen, UK) and seeded on Poly-D-Lysin/Laminin (Sigma-Aldrich, MO, USA) pre-coated dishes to form the monolayer mNSCs culture.

The 33Qn1-derived hiPSCs line was applied in this study, using non-integrating reprogramming vectors differentiated and cultured as described previously [18].

### EF stimulation on NSCs and hiPSCs

Monolayer NSCs (in neurobasal medium containing with L-glutamine and Laminin) or hiPSCs were subjected to a physiological direct current EF at 100 mV/mm, for 2 h per day, 1–7 days, as described previously [11]. Both Cell viability and morphology were monitored during the EF stimulation to ensure a healthy cell status (Additional file 1: Figure S1). The treated cells were then used for the following signaling pathway and transplantation studies**.** Non-stimulated (NoEF) cells were used as a negative control. After the EF stimulation, the NSCs and iPSCs were collected for the following immunofluorescence, protein and mRNA expression detects.

### Electrophysiological recordings

Voltage and current recordings were performed as described previously [18], using conventional patch-clamp in the whole-cell configuration [39] employing an Axopatch 200B amplifier interfaced to a computer running pClamp 9 using a Digidata 1322A A/D interface (Molecular Devices, Sunnyvale, CA, USA.).

### Spinal cord impact surgery and NSCs transplantation

The spinal cord impact surgery was performed on C57BL/6 mice 7 days before NSCs transplantation. The mice were anesthetized with 2% (v/v) isoflurane in oxygen. The laminectomy surgery was administrated to expose the spinal cord at T8-9. The mice then received impact injury at the exposed portion using an Infinite Horizon Impactor (Precision Systems and Instrumentation; Lexington, KY, USA). The diameter of the impactor top tip was 0.7 mm. The impacting parameters were set at 50 kilodynes for force and 90° for angle. The spinal, muscle and skin lesions were then sutured up, following the impactor was withdrew 1 s after the impact. For analgesic regimen, the mice received subcutaneous Carprofen at 2 mg/kg at the time of surgery. Following surgery, all mice received extensive post-operative care including bladder express twice a day until reflex voiding of the bladder was re-established.

The NSCs transplantation was performed 7 days after the SCI surgery. For stem cell injection, the mice received anesthesia of 2% (v / v) isoflurane in oxygen. The spinal cord lesion was re-opened to expose the impact point. The NSCs were injected with a microsyringe (Hamilton, USA) coupled with a needle (0.D. × I.D.: 0.31 mm × 0.16 mm, Hamilton, USA). Two microliter of cell suspension (10^8^ cells/mL) was intra-spinally injected at both 1 mm rostral (1 µL) and caudal (1 µL) of the impact point, in total of 200,000 cells per receiving mouse. The injecting speed was controlled at 0.5 µL/min by a Quintessential Stereotaxic Injector (Hamilton, USA). The needle tip was maintained inside the tissue 1 min after each injection to avoid liquid reflux. The spinal, muscle and skin lesions were then sutured up. For the analgesic regimen, the mice received subcutaneous Carprofen at 2 mg/kg at the time of surgery. Following injury, all mice received extensive post-operative care twice daily for one week.

### Spinal cord tissue fixation and sectioning

The euthanized mice received a cardiac perfusion and fixation with ice-cold 0.1 M PB solution and 4% paraformaldehyde (PFA, Sigma-Aldrich, MO, USA). The spinal cord was then dissected and incubated in 4% PFA at 4 °C for 3 days before transferring into a 30% (v/v) sucrose solution at 4° C for another 3 days. After cryoprotection, the spinal cord was frozen in liquid N_2_ and transversally sectioned in 20 µm intervals with a cryostat microtome. To track the transplanted NSCs and the neurogenesis post SCI, previously published methods were used [40]. Specifically, transversal spinal cord sections were collected between T7-T10 for the following immunofluorescence.

### Immunofluorescence

As described in our previous study [38], the NSCs and spinal cord tissue was fixed in 4% PFA and permeabilized with 0.1% Triton X-100 (Sigma-Aldrich, MO, USA). After blocking the non-specific proteins with 3% BSA-PB solution, the cells were then incubated with the primary antibodies: MAP2 (1:200, #4542S, Cell Signalling Technology, MA, USA), GFAP (1:200, #3670S, Cell Signalling Technology, MA, USA), βIII-tubulin (1:200, #5568, Cell Signalling Technology, MA, USA), Synapsin (1:200, #MA5-31919, ThermoFisher, USA), Synaptophys (1:200, #ab8049, Abcam, UK), Ki67 (1:1000, 9129S, Cell Signaling Technology, MA, USA) and Alexfluor 594/488-conjugated secondary antibodies (1:1000, Invitrogen, UK). DAPI was applied to label the nucleus. The fluorescence data was collected and analyzed with DeltaVision Elite Deconvolution/TIRF microscope system and laser-scanning confocal microscopy (Leica SP8 STED 3X microscope with 20X and 63 × 1.4 NA objectives).

### Quantification of transplanted NSCs in spinal cord

The immunopositive cell counting and neurite process measurement were performed with ImageJ software, for analysis of neurogenesis in the T7-T10 spinal cord. The EGFP + /Ki67 + , EGFP + /MAP2 + , EGFP + /GFAP + NSCs cell count and EGFP + neurite process measurements were respectively performance with 12 section intervals (20 μm/section, total thickness: 240 µm, from T7-T10) using the 20X and 40X objectives. The cell number and process length in T7-T10 of each animal were calculated and averaged to obtain the group mean and standard deviation.

### Western blotting

The cell lysate was collected after treatments for Wester blotting. The lysate samples were subjected onto 4–12% SDS-PAGE gel for electrophoresis as previously described [38]. Protein bands were then transblotted onto PVDF membrane (0.2 µm, ThermoFisher, USA), followed by blocking in 5% BSA-TBST buffer in room temperature for 1 h. The membrane was then incubated with primary antibodies targeting at: Akt (1:1000, #9272S, Cell Signaling Technology, MA, USA), p-Akt (1:1000, #9271S, Cell Signaling Technology, MA, USA), GSK-3β (1:1000, #9315S, Cell Signaling Technology, MA, USA), p-GSK-3β (Ser9, 1:1000, #5558S, Cell Signaling Technology, MA, USA), β-catenin (1:1000, # C2206-0.2ML, Sigma, USA), NeuroD1 (1:1000, #4373S, Cell Signaling Technology, MA, USA), GAPDH (1:1000, #2118 s, Cell Signaling Technology, MA, USA), overnight at 4 °C, followed by incubation with HRP-conjugated secondary antibodies (Cell Signaling Technology, MA, USA), at room temperature for 1 h.. GAPDH (1:1000, #2118S, Cell Signaling Technology, MA, USA) was detected as a loading control. The antigen–antibody complexes were then detected with an ECL reagent kit (#10455145, Thermo Scientific). The protein analysis was performed with *ImageJ* software.

### Real-time PCR analysis

Total mRNA of each group was extracted from the treated NSCs or spinal cord tissue RNeasy Mini Kit (#74104, QIAGEN, USA) according to the manufacturer's directions. The total yield of RNA per extraction was calculated using a NanoVue spectrophotometer (GE Healthcare) to measure the absorbance at 260 nm. The exacted mRNA in 2000 ng was then used to synthesis cDNA using MMLV reverse transcriptase (Promega), following a reported protocol [38]. The real-time PCR reactions with the cDNA were performed by ABI ViiA7 Fast sequence detection system (Advanced Biosystems) and the amplifications were detected using SYBR Green PCR Master Mix (PrimerDesign, UK). The sequences of the primers were shown in Table 1. The cycling conditions was set as: an initial denaturation step of 95 °C for 2 min followed by 40 cycles of 15 s denaturation (95 °C) and 1-min annealing/elongation at 60 °C. The mRNA expression of GAPDH was set as internal control and relative to a control sample (untreated cells). The relative quantification in gene expression was determined using the 2^−ΔΔCt^ method [41].

**Table 1:** List of the primers used in this study

Primer	Sequence 5ʹ-3ʹ	Accession Number
NES forward	CCAAAGAGGTGTCCGATCATC	NM_016701.3
NES reverse	CTCCCTTCTTCTTCATCAGCATCT	
NeuroD1 forward	AAGCCATGAATGCAGAGGAGGACT	NM_010894.3
NeuroD1 reverse	AGCTGCAGGCAGCCGGCGACC	
MAP2 forward	TGACACTTGGGACCTGGACGAGTAT	NM_008632.2
MAP2 reverse	ACACCACTTCTTCAACCAACGCTCA	
GFAP forward	CAACTTTGCACAGGACCTCGGCACCCT	NM_001131020.1
GFAP reverse	GGCGGCGATAGTCGTTAGCTTCGTGCT	
GAPDH forward	AGGTCGGTGTGAACGGATTTG	NM_008084.3
GAPDH reverse	TGTAGACCATGTAGTTGAGGTCA	

### β-catenin knock-down

RNAi was applied to knock down the protein expression of β-catenin, following manufacturer’s instructions of β-catenin siRNA (targeting at CTNNB1, mouse specific, Cell Signalling Technology, MA, USA) and Lipofectamine 2000 (Life technologies, CA, USA). Briefly, the monolayer culture of mNSCs was cultured in medium containing Lipofectamine 2000-siRNA complex for 5 h, and then subjected to EF/noEF treatment. More transfected cells were cultured in medium for 24, 48 and 72 h to investigate the efficiency of transfection with immunofluorescence and western blotting.

### Statistics

Quantitative data were analyzed using Clampfit 10.3 (Molecular Devices), Microsoft Excel, Microcal Origin 6.0 and GraphPad Prism 8. BBB score and patch-clamp data were expressed as mean ± S.E.M; other data was presented as means ± S.D. The statistical significance was evaluated by one-way ANOVA for each group, followed by a Tukey’s Honestly Significant Difference *post-hoc* analysis; and by a two-way ANOVA test for the analysis when including three or more groups, followed by a Dunnett's multiple comparisons test.

## Supplementary Information

**Additional file 1**: **Table S1**. Proportions and percentages of Q33n1 hiPSC-derived NPCs which demonstrated each of the different types of induced action potentials for electric field (EF) stimulated and non-stimulated control (NoEF) at weeks 1-3.* None* = no significant voltage excursions from baseline; *Attempting Single* = voltage excursions which do not overshoot 0 mV; *Single* = one excursion only, which overshoots 0 mV; *Attempting Train* = several excursions, but only one which overshoots 0 mV; *Train* = several excursions, with more than one which overshoots 0 mV. **Table S2**. Analysis of passive and active parameters of induced action potentials of Q33n1 hiPSC-derived NPSs stimulated with an electric field (EF) and nonstimulated (NoEF) at weeks 1-3. * *P *< 0.05, *** *P *< 0.001. *-*** were considered as significantly different from correspondent values of NoEF Q33n1 hiPSC-derived groups of NPSs. Abbreviations: Membrane potential (Vm), input resistance (Rin), whole cell capacitance (Cp), I Na_max_ and I K_max_ are Na^+^ and K^+^ currents, respectively. n: cell number. **Figure S1**. Safety of EF stimulation on NSCs. A. The MTT assay demonstrated lower absorbance with EF stimulation on NSCs for 4 to 7 days. * *P *< 0.05 was considered as significantly different between EF and NoEF groups. B. The morphology observation displayed healthy and active status of both NSCs with and without EF stimulation. But the smaller cell population and longer axon process were detected in the EF stimulated group. Scale bar: 20 µm. **Figure S2**. EF promoted neuronal differentiation of hiPSC-derived NPCs. A-B. The EF stimulation for 14 days *in vitro* induced synaptophysin and βIII-tubulin up-regulation in hiPSC-derived NPCs. Scale bar: 20 µm. C. Pie charts displaying percentage and proportion of Q33n1 hiPSC-derived neurons, EF stimulated (upper panel) and noEF (lower panel), cultured *in vitro *for 1-3 weeks exhibiting: no activity - *Quiet* (red), *Attempting* activity - (amber) or *Spontaneous* activity (green). D. Exemplar traces of membrane potential of Q33n1 hiPSC-derived neurons which demonstrates each different type of activity (Quiet, Attempting and Spontaneous). E. Mean ± S.E.M. values of membrane potential of EF stimulated (filled squares) and noEF (empty circles) Q33n1 hiPSC-dervied neurons at weeks 1-3 of culturing *in vitro. *The synaptophysin+ and βIII-tubulin+ positive cell count and percentage data were presented as mean ± SD. * *P* < 0.05 and ** *P* < 0.01 were considered as significantly different between EF and NoEF groups. **Figure S3**. Effect of EF treatment on voltage-gated Na^+^ current activation and inactivation characteristics during hiPSC differentiation (A). Exemplar family of whole cell currents (upper) during the activation/inactivation voltage protocol (lower). Inset (right) illustrate Na^+^ currents. Peak Na^+^ current activation and inactivation levels are shown by the red and blue arrows, respectively. (B-G). Mean activation and inactivation curves of whole-cell Na^+^ currents recorded in EF treated Q33n1-hiPSC derived neuronscultured *in vitro *at weeks 1 -3 (B, D, F) and noEF control (C, E, G). Activation curves are depicted by the filled squares and inactivation curves are shown by the empty circles. On each panel individual Vm values (filled upward triangles) and mean Vm values (red arrow on *abscissa*) are also shown. Voltages of half maximal action (V_a_50) and half maximal inactivation (V_i_50) are also indicated in each panel, along with h factors, mean crossing points (downward arrows) and number of cells (n) recorded. **Figure S4**. The differentiating potential of PI3Kγ ^-/-^and PI3Kγ^KD/KD^ NSCs. The PI3Kγ ^-/-^and PI3Kγ^KD/KD^ NSCs were plated on slides for monolayer culture, differentiation induction and immunofluorescence. For the differentiation induction, 0.1% FBS was added to the culture medium for 7 days. A. MAP2 and GFAP positive cells in the PI3Kγ ^-/-^and PI3Kγ^KD/KD^ NSCs with 7-day differentiation induction. B. Cellcount ratio of MAP2+ cells from the induced PI3Kγ ^-/-^and PI3Kγ^KD/KD^ NSCs. C. Cellcount ratio of GFAP+ cells from the induced PI3Kγ ^-/-^and PI3Kγ^KD/KD^ NSCs. Scale bar: 20 µm. * *P *< 0.05 was considered as significantly different between EF and NoEF groups.

## Data Availability

The data that support the findings of this study are available on request from the corresponding authors, Q.L. and B.S.
