# Supplementary material for: Electric field stimulation boosts neuronal differentiation of neural stem cells for spinal cord injury treatment via PI3K/Akt/GSK-3β/β-catenin activation
Source: Cell Biosci. 2023 Jan 9;13:4. doi: 10.1186/s13578-023-00954-3 (PMC9830810; doi:10.1186/s13578-023-00954-3)
Supplement: Supplementary file 1 — Additional file 1: Table S1. Proportions and percentages of Q33n1 hiPSC-derived NPCs which demonstrated each of the different types of induced action potentials for electric field (EF) stimulated and non-stimulated control (NoEF) at weeks 1-3. None = no significant voltage excursions from baseline; Attempting Single = voltage excursions which do not overshoot 0 mV; Single = one excursion only, which overshoots 0 mV; Attempting Train = several excursions, but only one which overshoots 0 mV; Train = several excursions, with more than one which overshoots 0 mV. Table S2. Analysis of passive and active parameters of induced action potentials of Q33n1 hiPSC-derived NPSs stimulated with an electric field (EF) and nonstimulated (NoEF) at weeks 1-3. * P < 0.05, *** P < 0.001. *-*** were considered as significantly different from correspondent values of NoEF Q33n1 hiPSC-derived groups of NPSs. Abbreviations: Membrane potential (Vm), input resistance (Rin), whole cell capacitance (Cp), I Namax and I Kmax are Na+ and K+ currents, respectively. n: cell number. Figure S1. Safety of EF stimulation on NSCs. A. The MTT assay demonstrated lower absorbance with EF stimulation on NSCs for 4 to 7 days. * P < 0.05 was considered as significantly different between EF and NoEF groups. B. The morphology observation displayed healthy and active status of both NSCs with and without EF stimulation. But the smaller cell population and longer axon process were detected in the EF stimulated group. Scale bar: 20 µm. Figure S2. EF promoted neuronal differentiation of hiPSC-derived NPCs. A-B. The EF stimulation for 14 days in vitro induced synaptophysin and βIII-tubulin up-regulation in hiPSC-derived NPCs. Scale bar: 20 µm. C. Pie charts displaying percentage and proportion of Q33n1 hiPSC-derived neurons, EF stimulated (upper panel) and noEF (lower panel), cultured in vitro for 1-3 weeks exhibiting: no activity - Quiet (red), Attempting activity - (amber) or Spontaneous activity (green). D. Exempla [file 13578_2023_954_MOESM1_ESM.docx]

**Additional file Results**

**Physiological EF stimulation did no harm to NSCs**

The cultured neural stem cells (NSCs) were subjected to physiological electric field (EF) stimulation at 100 mV/mm for 1-7 days. To verify the effect on cell viability and activity, the MTT (3-(4,5-dimethylthiazol-2-yl)-2,5-diphenyltetrazolium bromide) assay and morphology observation was performed prior to the further investigation. Compared to the NoEF treated control NSCs, the EF stimulated NSCs demonstrated significant lower MTT absorbance value with 4 to 7 days (Figure S1A). While both cells were observed in healthy and active status (Figure S1B). We explained the lower MTT absorbance value by EF stimulation as a result that EF stimulation promoted differentiation, especially neuronal differentiation rather than proliferation of NSCs which led to smaller cell population from 4 to 7 days with the EF stimulation than those with NoEF treatment (from cell morphology observation in Figure S1B).

**EF promoted neuronal differentiation of hiPSCs-derived NPCs**

To verify the EF promoted neuronal differentiation, we administered the EF stimulation on 33Qn1-derived human induced pluripotent stem cells (hiPSC), which demonstrated a significant effect in promoting neuronal differentiation and maturation. According to immunofluorescence data EF stimulated hiPSCs-derived neural precursor cells (NPCs) expressed significantly increased synaptophysin and βIII-tubulin, markers of neuronal differentiation (Figure S2A-S2C).

The 33Qn1 hiPSC-derived NPCs stimulated by EF also displayed a significantly augmented proportion of neurons showing spontaneous electrical activity, a neuronal maturation signature, compared to the control NoEF treated 33Qn1 hiPSC-derived NPCs from week 1 through 3 (Figure S3). On week 1 with EF stimulation yielded in 5% (1/21) of neurons which were coded as Spontaneous, 38% (8/21) were considered as Attempted and 57% (12/21) as Quiet. In the control NoEF 33Qn1 hiPSC-derived NPCs, there were no Spontaneous cells, only 18% (4/22) of Attempted and 82% (18/22) of Quiet. On week 2, EF stimulated 33Qn1 hiPSC-derived NPCs demonstrated 48% (9/19) of Spontaneous, 26% (5/19) of Attempted and 26% (5/19) of Quiet cells. While the control NoEF 33Qn1 hiPSC-derived NPCs demonstrated only 17% (4/24) of Spontaneous, 21% (5/24) of Attempted and 63% (15/24) of Quiet cells. On week 3, EF stimulated 33Qn1 hiPSC-derived NSCs demonstrated 68% (15/22) of Spontaneous neurons and 32% (7/22) Attempted, but no Quiet cells. Whereas the control NoEF 33Qn1 hiPSC-derived NPCs demonstrated 50% (11/22) of Spontaneous, 36% (8/22) of Attempted and 14% (3/22) of Quiet cells (Figure S3A-S3B).

Besides the augmented neuronal activity, EF stimulation correspondently increased the mean resting membrane potential (Vm) of 33Qn1 hiPSC-derived NPCs: on week 1 it was -39.5 ± 2.7 mV (n = 21), significantly different from Vm of the control NoEF 33Qn1 hiPSC-derived NPCs: -32.2 ± 2.0 mV (n = 22), *P* < 0.05. On week 2 the difference increased further: the Vm of EF stimulated 33Qn1 hiPSC-derived NPCs was -44.9 ± 2.2 mV (n = 19) vs. the control NoEF 33Qn1 hiPSC-derived neurons -33.3 ± 2.2 mV (n = 22), *P* < 0.001. On week 3 Vm of 33Qn1 hiPSC-derived neurons was -51.9 ± 1.9 mV (n = 22) vs. the control NoEF cells -44.5 ± 2.7 mV (n = 22), *P* < 0.05 (Figure S4 and Table S2).

An important parameter that characterises functional and morphological maturation of the neurons is the cell capacitance (Cp) because it correlates with the cell square surface, cell size and the complexity of the neuronal outgrowth. Cp displayed significant differences at least during the first two weeks of neuronal differentiation of 33Qn1 hiPSC-derived neurons. On week 1 the Cp of EF 33Qn1 hiPSC-derived neurons was 13.8 ± 1.2 pF (n = 14), whereas in the control noEF cells it was only 9.9 ± 1.1 pF (n = 13), P < 0.05. On week 2 Cp of EF 33Qn1 hiPSC-derived neurons was 21.2 ± 2.0 mV (n = 16) vs. that in the control NoEF cells 15.9 ± 1.4 mV (n = 18), *P* < 0.05. On week 3, Cp values of EF and NoEF 33Qn1 hiPSC-derived neurons were 30.0 ± 2.7 mV (n = 19) and 23.6 ± 2.5 mV (n = 16), respectively. The data at this timepoint however were considered as not significantly different (Table S2).

Other parameters of induced action potentials presented in Table S2, such as threshold (mV), overshoot (mV), after hyperpolarization (mV), amplitude (mV), depolarization rate (V/s), repolarization rate (V/s), half width (ms) and maximal values of Na^+^ and K^+^ currents I Na_max_ (nA), I K_max_ (nA), respectively, displayed certain trends of augmented maturation of EF stimulated 33Qn1 hiPSC-derived neurons compared to the control NoEF neurons, however no significant difference was detected between the groups.

Notwithstanding the fact that EF stimulation only slightly increased absolute Na^+^ and K^+^ currents on weeks 2 and 3, their current densities (pA/pF) displayed no difference compared to the NoEF cells. In the same time activation/inactivation profiles of the voltage-activated Na^+^ currents in EF stimulated 33Qn1 hiPSC-derived neurons exhibited larger availability windows with high G/Gmax maxima and a higher proportion of the neurons with Vm values falling within those windows when compared to NoEF neurons (FigureS4).

To summarize, these data suggest that EF stimulation enhances functional maturation of 33Qn1 hiPSC-derived neurons by two major biophysical enhancements: by hyperpolarizing the cells to withdraw inactivation of voltage-gated Na^+^ channels which enables their higher spontaneous activity, and by increasing the Na^+^ current availability, to facilitate regenerative action potential Train activity.

**Additional file Tables**

**Table S1.** **Proportions and percentages of Q33n1 hiPSC-derived NPCs which demonstrated each of the different types of induced action potentials for electric field (EF) stimulated and non-stimulated control (NoEF) at weeks 1-3**. *None* = no significant voltage excursions from baseline; *Attempting Single* = voltage excursions which do not overshoot 0 mV; *Single* = one excursion only, which overshoots 0 mV; *Attempting Train* = several excursions, but only one which overshoots 0 mV; *Train* = several excursions, with more than one which overshoots 0 mV.

| **Type of activity** | | **Week 1** | | **Week 2** | | **Week 3** | |
| --- | --- | --- | --- | --- | --- | --- | --- |
|  |  | **Proportion** | **%** | **Proportion** | **%** | **Proportion** | **%** |
| ***EF*** | None | 0/17 | 0 | 0/17 | 0 | 0/19 | 0 |
|  | Attempting single | 0/17 | 0 | 0/17 | 0 | 0/19 | 0 |
|  | Single | 3/17 | 18 | 1/17 | 6 | 1/19 | 5 |
|  | Attempting train | 7/17 | 41 | 1/17 | 6 | 0/19 | 0 |
|  | Train | 7/17 | 41 | 15/17 | 88 | 18/19 | 95 |
| ***NoEF*** | None | 0/17 | 0 | 0/19 | 0 | 0/20 | 0 |
|  | Attempting single | 1/17 | 6 | 0/19 | 0 | 0/20 | 0 |
|  | Single | 10/17 | 59 | 5/19 | 26 | 2/20 | 10 |
|  | Attempting train | 3/17 | 18 | 2/19 | 11 | 1/20 | 5 |
|  | Train | 3/17 | 18 | 12/19 | 63 | 17/20 | 85 |

**Table S2. Analysis of passive and active parameters of induced action potentials of Q33n1 hiPSC-derived NPSs stimulated with an electric field (EF) and nonstimulated (NoEF) at weeks 1-3**. * *P* < 0.05, *** *P* < 0.001. *-*** were considered as significantly different from correspondent values of NoEF Q33n1 hiPSC-derived groups of NPSs. Abbreviations: Membrane potential (Vm), input resistance (Rin), whole cell capacitance (Cp), I Na_max_ and I K_max_ are Na^+^ and K^+^ currents, respectively. n: cell number.

|  | | | **Week 1** | | | **Week 2** | | | **Week 3** | | |
| --- | --- | --- | --- | --- | --- | --- | --- | --- | --- | --- | --- |
|  |  |  | **Mean** | **SEM** | **n** | **Mean** | **SEM** | **n** | **Mean** | **SEM** | **n** |
| ***EF*** | ***Passive*** | Vm (mV) | -39.0* | 2.7 | 21 | -44.9*** | 2.2 | 19 | -51.9* | 1.9 | 22 |
|  |  | Rin (GΩ) | 1.4 | 0.2 | 17 | 0.9 | 0.1 | 17 | 0.7 | 0.1 | 19 |
|  |  | Cp (pF) | 13.8* | 1.2 | 14 | 21.2* | 2.0 | 16 | 30.0 | 2.7 | 19 |
|  | ***Spike Analysis and Currents*** | Threshold (mV) | -34.2 | 1.6 | 17 | -35.8 | 2.0 | 17 | -38.3 | 1.1 | 19 |
|  |  | Overshoot (mV) | 18.5 | 3.2 | 17 | 28.2 | 3.1 | 17 | 32.2 | 3.1 | 19 |
|  |  | Afterhyperpo-larization (mV) | -48.9 | 1.8 | 17 | -57.7 | 1.0 | 17 | -61.4 | 2.2 | 19 |
|  |  | Amplitude (mV) | 67.4 | 4.2 | 17 | 86.0 | 3.4 | 17 | 93.7 | 4.6 | 19 |
|  |  | Depolarization rate (V/s) | 41.6 | 5.5 | 17 | 62.4 | 6.8 | 17 | 95.5 | 9.5 | 19 |
|  |  | Repolarization rate (V/s) | -17.6 | 2.1 | 17 | -33.1 | 3.4 | 17 | -49.4 | 5.5 | 19 |
|  |  | Half width (ms) | 4.3 | 0.3 | 17 | 3.0 | 0.2 | 17 | 2.2 | 0.1 | 19 |
|  |  | I Na_max_ (nA) | -1.6 | 0.2 | 13 | -3.8 | 0.6 | 16 | -6.2 | 0.6 | 19 |
|  |  | I K_max_ (nA) | 2.1 | 0.3 | 13 | 3.2 | 0.4 | 16 | 6.0 | 0.6 | 19 |
| ***NoEF*** | ***Passive*** | Vm (mV) | -32.2 | 2.0 | 22 | -33.3 | 2.2 | 22 | -44.5 | 2.7 | 22 |
|  |  | Rin (GΩ) | 1.1 | 0.1 | 17 | 0.9 | 0.1 | 20 | 0.6 | 0.1 | 20 |
|  |  | Cp (pF) | 9.9 | 1.1 | 13 | 15.9 | 1.4 | 18 | 23.6 | 2.5 | 16 |
|  | ***Spike Analysis and Currents*** | Threshold (mV) | -33.4 | 1.9 | 16 | -36.9 | 1.8 | 19 | -38.3 | 4.4 | 20 |
|  |  | Overshoot (mV) | 11.5 | 2.6 | 16 | 24.3 | 3.7 | 19 | 24.2 | 2.8 | 20 |
|  |  | Afterhyperpo-larization (mV) | -53.0 | 1.8 | 16 | -56.2 | 0.9 | 19 | -61.1 | 0.9 | 20 |
|  |  | Amplitude (mV) | 64.5 | 3.6 | 16 | 80.5 | 4.0 | 19 | 85.3 | 2.9 | 20 |
|  |  | Depolarization rate (V/s) | 33.1 | 4.7 | 16 | 57.0 | 6.5 | 19 | 79.2 | 6.3 | 20 |
|  |  | Repolarization rate (V/s) | -16.8 | 2.6 | 16 | -27.5 | 3.2 | 19 | -45.4 | 3.7 | 20 |
|  |  | Half width (ms) | 5.0 | 0.5 | 16 | 3.5 | 0.3 | 19 | 2.2 | 0.1 | 20 |
|  |  | I Na_max_ (nA) | -2.0 | 0.5 | 11 | -3.3 | 0.6 | 17 | -5.6 | 0.6 | 16 |
|  |  | I K_max_ (nA) | 2.2 | 0.2 | 11 | 2.5 | 0.3 | 17 | 4.8 | 0.5 | 15 |

**Additional file Figures**

**Figure S1**

**
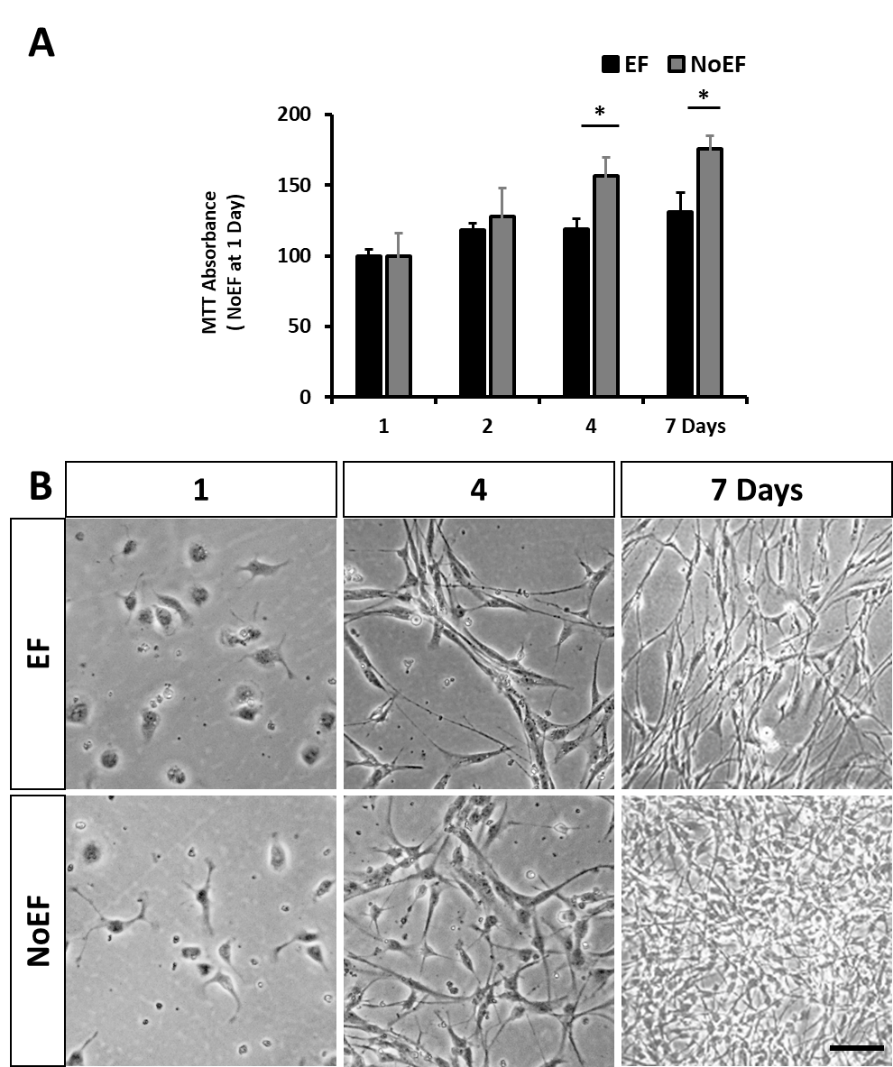
**

**Figure S1. Safety of EF stimulation on NSCs. A.** The MTT assay demonstrated lower absorbance with EF stimulation on NSCs for 4 to 7 days. * *P* < 0.05 was considered as significantly different between EF and NoEF groups. **B.** The morphology observation displayed healthy and active status of both NSCs with and without EF stimulation. But the smaller cell population and longer axon process were detected in the EF stimulated group. Scale bar: 20 µm.

**Additional file Figure S2**

**
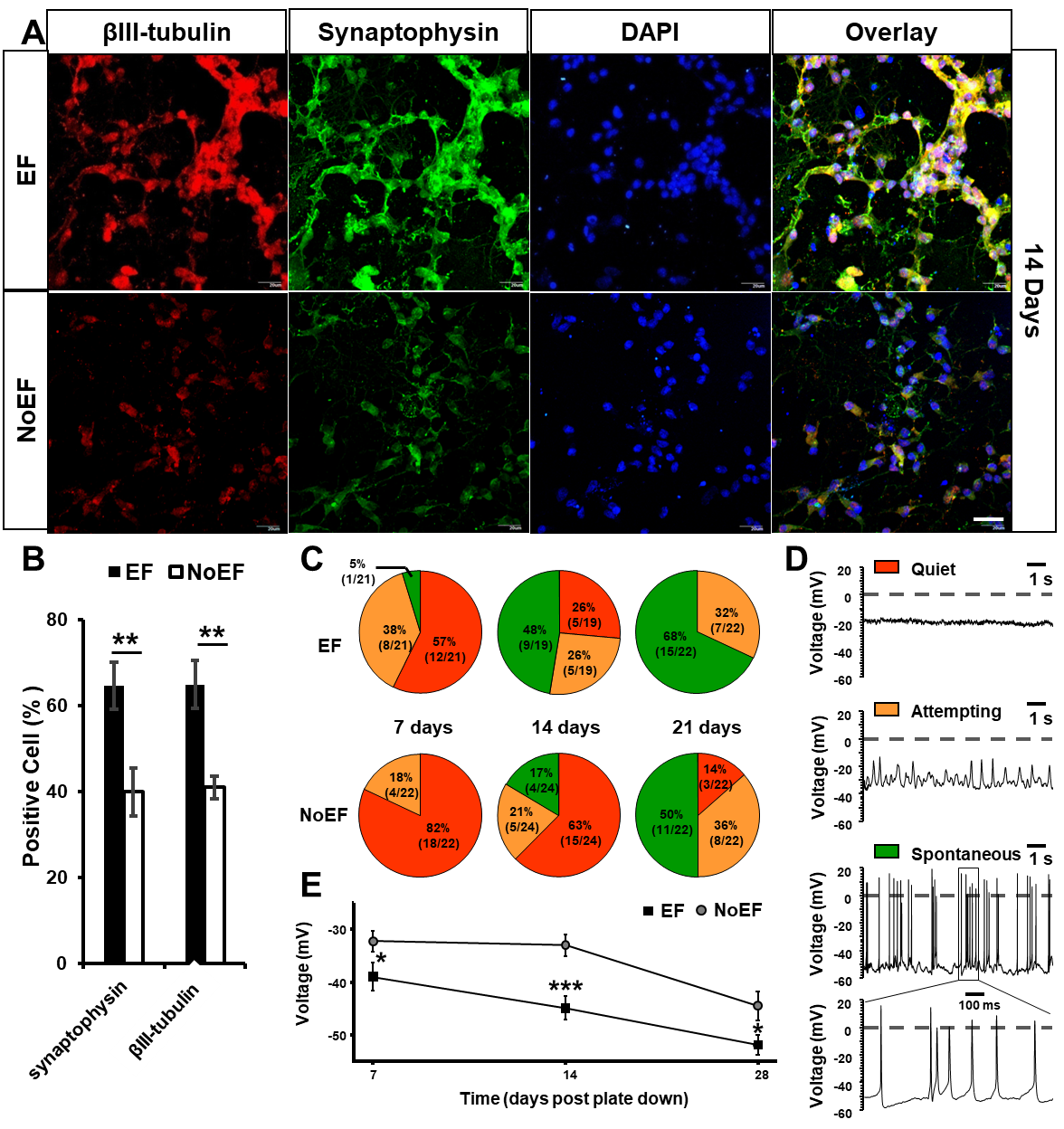
**

**Figure S2. EF promoted neuronal differentiation of hiPSC-derived NPCs.** **A-B.** The EF stimulation for 14 days *in vitro* induced synaptophysin and βIII-tubulin up-regulation in hiPSC-derived NPCs. Scale bar: 20 µm. **C.** Pie charts displaying percentage and proportion of Q33n1 hiPSC-derived neurons, EF stimulated (upper panel) and noEF (lower panel), cultured *in vitro* for 1-3 weeks exhibiting: no activity - *Quiet* (red), *Attempting* activity - (amber) or *Spontaneous* activity (green). **D.** Exemplar traces of membrane potential of Q33n1 hiPSC-derived neurons which demonstrates each different type of activity (Quiet, Attempting and Spontaneous). **E.** Mean ± S.E.M. values of membrane potential of EF stimulated (filled squares) and noEF (empty circles) Q33n1 hiPSC-dervied neurons at weeks 1-3 of culturing *in vitro.* The synaptophysin+ and βIII-tubulin+ positive cell count and percentage data were presented as mean ± SD. * *P* < 0.05 and ** *P* < 0.01 were considered as significantly different between EF and NoEF groups.

**Additional file Figure S3**


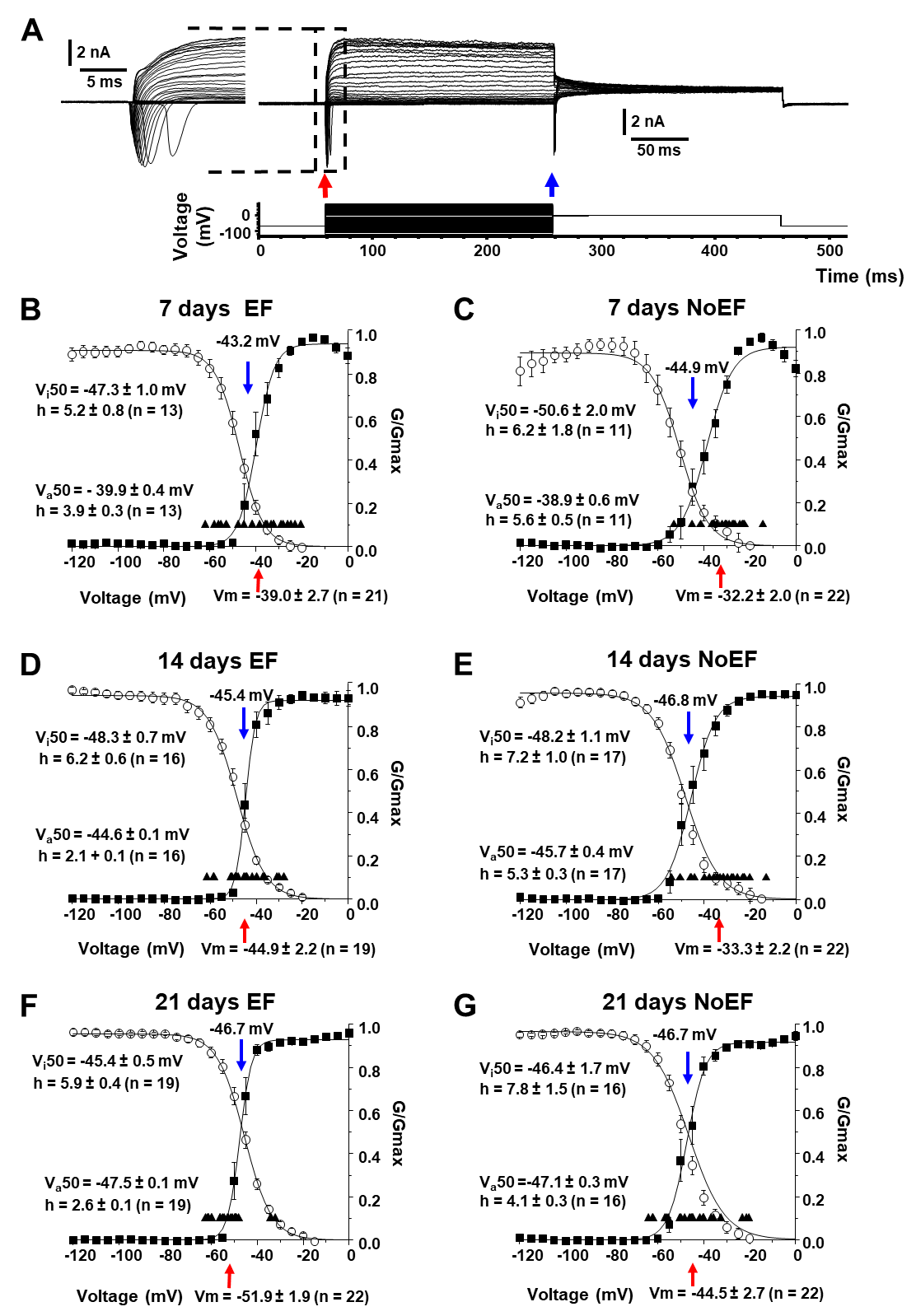


**Figure S3. Effect of EF treatment on voltage-gated Na^+^ current activation and inactivation characteristics during hiPSC differentiation** (A). Exemplar family of whole cell currents (upper) during the activation/inactivation voltage protocol (lower). Inset (right) illustrate Na^+^ currents. Peak Na^+^ current activation and inactivation levels are shown by the red and blue arrows, respectively. (B-G). Mean activation and inactivation curves of whole-cell Na^+^ currents recorded in EF treated Q33n1-hiPSC derived neuronscultured *in vitro* at weeks 1 -3 (B, D, F) and noEF control (C, E, G). Activation curves are depicted by the filled squares and inactivation curves are shown by the empty circles. On each panel individual Vm values (filled upward triangles) and mean Vm values (red arrow on *abscissa*) are also shown. Voltages of half maximal action (V_a_50) and half maximal inactivation (V_i_50) are also indicated in each panel, along with h factors, mean crossing points (downward arrows) and number of cells (n) recorded.

**Additional file Figure S4**


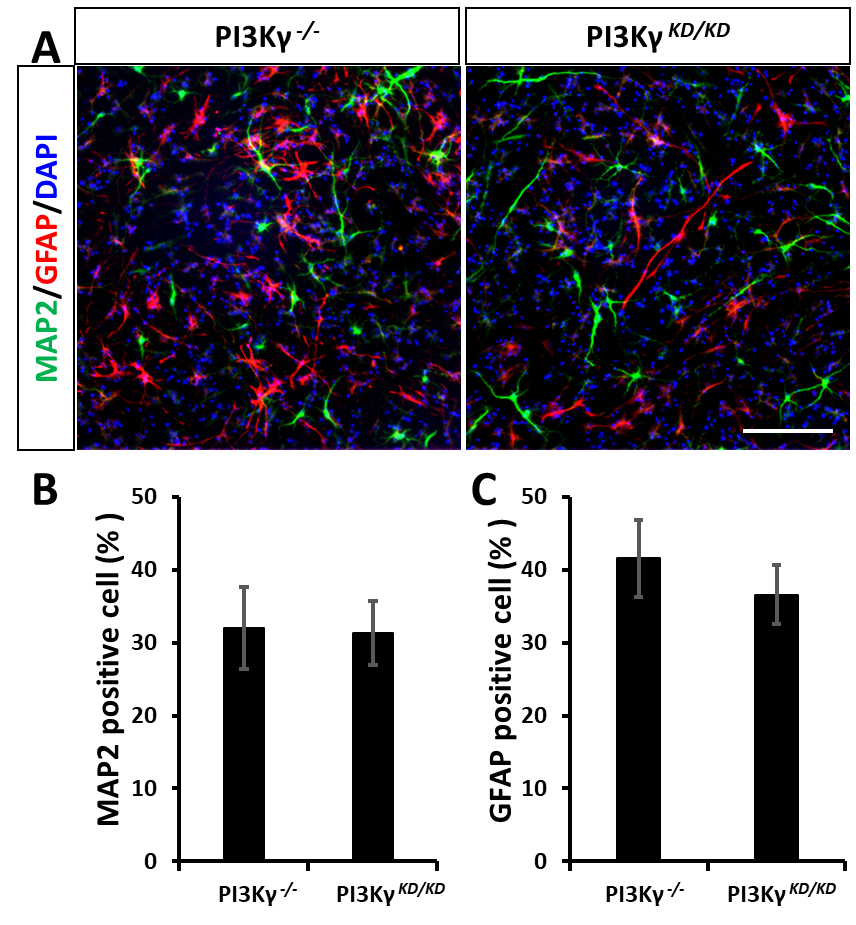


**Figure S4. The differentiating potential of PI3Kγ ^-/-^and PI3Kγ^KD/KD^ NSCs.** The PI3Kγ ^-/-^and PI3Kγ^KD/KD^ NSCs were plated on slides for monolayer culture, differentiation induction and immunofluorescence. For the differentiation induction, 0.1% FBS was added to the culture medium for 7 days. **A.** MAP2 and GFAP positive cells in the PI3Kγ ^-/-^and PI3Kγ^KD/KD^ NSCs with 7-day differentiation induction. **B.** Cellcount ratio of MAP2+ cells from the induced PI3Kγ ^-/-^and PI3Kγ^KD/KD^ NSCs. C. Cellcount ratio of GFAP+ cells from the induced PI3Kγ ^-/-^and PI3Kγ^KD/KD^ NSCs. Scale bar: 20 µm. * *P* < 0.05 was considered as significantly different between EF and NoEF groups.
